# Enhanced Stability of Iridium Nanocatalysts via Exsolution for the CO_2_ Reforming of Methane

**DOI:** 10.1021/acsanm.3c04126

**Published:** 2023-12-01

**Authors:** Eleonora Calì, Shailza Saini, Gwilherm Kerherve, William S. Skinner, Ian S. Metcalfe, David J. Payne, Kalliopi Kousi

**Affiliations:** † Department of Applied Science and Technology, Politecnico di Torino, Corso Duca degli Abruzzi, 24, Turin 10129, Italy; ‡ Department of Materials, Imperial College London, Exhibition Road, London SW7 2AZ, United Kingdom; § School of Chemistry and Chemical Engineering, University of Surrey, Guildford GU2 7XH, United Kingdom; ∥ School of Engineering, Newcastle University, Merz Court, Newcastle upon Tyne NE1 7RU, United Kingdom; ⊥ Research Complex at Harwell, Harwell Science and Innovation Campus, Didcot, Oxfordshire OX11 0FA, United Kingdom

**Keywords:** exsolved nanocatalysts, dry methane reforming, CO_2_ conversion, coke resistance, environmental catalysis, perovskite oxides, greenhouse gases reformation

## Abstract

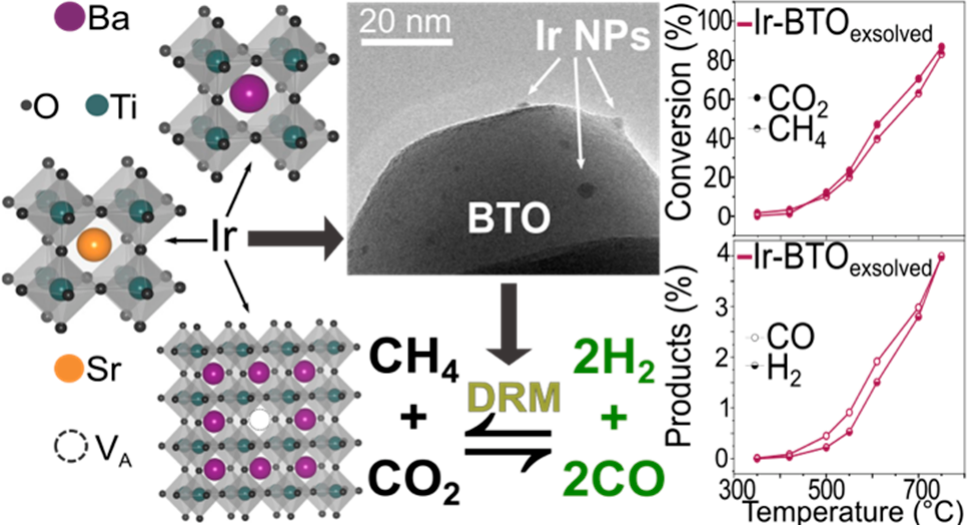

The reforming reactions of greenhouse gases require catalysts with high reactivity, coking resistance, and structural stability for efficient and durable use. Among the possible strategies, exsolution has been shown to demonstrate the requirements needed to produce appropriate catalysts for the dry reforming of methane, the conversion of which strongly depends on the choice of active species, its interaction with the support, and the catalyst size and dispersion properties. Here, we exploit the exsolution approach, known to produce stable and highly active nanoparticle-supported catalysts, to develop iridium-nanoparticle-decorated perovskites and apply them as catalysts for the dry reforming of methane. By studying the effect of several parameters, we tune the degree of exsolution, and consequently the catalytic activity, thereby identifying the most efficient sample, 0.5 atomic % Ir-BaTiO_3_, which showed 82% and 86% conversion of CO_2_ and CH_4_, respectively. By comparison with standard impregnated catalysts (e.g., Ir/Al_2_O_3_), we benchmark the activity and stability of our exsolved systems. We find almost identical conversion and syngas rates of formation but observe no carbon deposition for the exsolved samples after catalytic testing; such deposition was significant for the traditionally prepared impregnated Ir/Al_2_O_3_, with almost 30 mg_C_/g_sample_ measured, compared to 0 mg_C_/g_sample_ detected for the exsolved system. These findings highlight the possibility of achieving *in a single step* the mutual interaction of the parameters enhancing the catalytic efficiency, leading to a promising pathway for the design of catalysts for reforming reactions.

## Introduction

In 2022, global methane emissions from the energy sector alone were estimated to be approximately 135 million tons, only slightly lower than in the record year 2019.^1^ The energy sector is responsible for ∼35% of the anthropogenic emissions, and only 60% of total emissions are anthropogenic.^2^ It is worth noting that although methane is emitted in smaller quantities compared to carbon dioxide, it has a much higher warming potential per molecule. Additionally, we are currently emitting ∼38 billion tonnes of CO_2_ per year, a number that has decreased over the past few years, but the emissions have yet to reach their peak. With the increasing focus and effort toward reducing greenhouse gas emissions via their capture and reuse, the dry reforming of methane (DRM) (eq 1) has emerged as an interesting catalytic method to convert methane (CH_4_) and carbon dioxide (CO_2_) into carbon monoxide (CO) and hydrogen (H_2_).
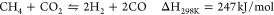
1

Through the Fischer–Tropsch synthesis (FTS), this produces syngas which can then be directly used to obtain high-value-added hydrocarbon-based fuels.^3,4^

These include olefins used in the packaging and food industries,^5^ as well as methanol, which is used as chemical in the pharmaceutical industry^6^ or sustainable fuels.^7^ Significant challenges involved in the DRM reaction are carbon deposition and sintering,^8,9^ caused by the high reaction temperatures required. Research in this area has been focused on the development of highly active, stable, and scalable catalysts resistant to deactivation. Several factors are known to influence the avoidance of such issues. These include, but are not limited to, the type of active metal employed, the catalyst surface area and particle size, the nature of the support, and its interaction with the catalyst nanoparticles. The most commonly studied metals for the DRM reaction are Ni and Co, mainly due to their low cost and high activity for this specific reaction.^10,10,11^ However, these are both highly prone to deactivation by sintering and carbon deposition.^3,8,12^ A partial solution to such issues would be in the use of noble metals, which have improved resistance to coke formation as well as activity and selectivity to syngas production.^13−15^ However, the high temperatures required to reach an appreciable activity and conversion often cause mobility and sintering of the active species on the support surface, especially when catalysts are developed via traditional synthesis methods such as vapor deposition or chemical infiltration.^16,17^ Therefore, a promising approach called “exsolution” has recently gathered interest due to its intrinsic characteristics of ease of synthesis and stability of the produced nanoparticles during catalytic application.^18−21^ In such a method, instead of having the catalytic nanoparticles deposited on the surface of the support, the catalytic species is incorporated into the structure of a host during initial materials synthesis, to then diffuse from the solid solution to the surface of the support via a reduction treatment (or through the application of an electrical potential or plasma treatment)^20,22−24^ in the form of “socketed” metallic nanoparticles. This results in the often observed exsolved materials’ unique stability to carbon deposition and sintering.^18,25^ Moreover, recent studies have also highlighted the role of the socketing, and consequent strain achieved between exsolved NPs and the support, in the enhanced catalytic activities measured for several processes, such as CO oxidation, CO_2_ reduction in solid oxide cells, and also CH_4_/CO_2_ conversion.^18,21,24,26−28^ Moreover, exploring the use of alkaline earth A-site perovskite oxides as supports might be beneficial due to their structural stability, the introduction of basic sites, which is known to promote surface reactions, and their lattice oxygen mobility, which could lead to the possible introduction of oxygen vacancies. These factors are all regarded as important in the activity observed for the DRM reaction.^17,29,30^

In this context, this work explores the use of a low amount of noble metal doping, specifically Ir, substituted in 0.5 atomic % (0.4 wt %) in perovskite structures for the dry reforming of methane reaction. A systematic study on the role of the NP formation thermal conditions, the structural defects introduced via A-site deficiency, and the changes in support chemistry by varying the A-site cation, has been carried out to understand the potential of such systems for the CO_2_ and CH_4_ conversion to syngas. In-depth characterization via X-ray photoelectron spectroscopy (XPS), scanning electron microscopy (SEM), X-ray diffraction (XRD), transmission electron microscopy (TEM), and scanning-transmission electron microscopy-energy dispersive X-ray spectroscopy (STEM-EDX) has elucidated the role of each investigated feature in the observed catalytic activity. Moreover, the activity and resistance to coking of our exsolved systems were studied by comparison with those of standard impregnated catalysts. Finally, the stability to coarsening of our exsolved catalysts appears to significantly outperform the highly active reference Ir/Al_2_O_3_, which shows instead extensive nanoparticle coalescence. These promising results show the high potential for such systems to be used for efficient CO_2_ conversion reactions, where the combined stability, efficiency, activity, and metal–support synergy can be achieved simultaneously in these low-Ir-doped exsolved Ba-oxide systems.

## Methods

The 0.5% Ir-substituted BaTiO_3_ and SrTiO_3_ samples were synthesized by a solid-state reaction method. Barium carbonate (BaCO_3_, Sigma-Aldrich, ≥99.9%) or strontium carbonate (SrCO_3_, Sigma-Aldrich, ≥99.9%), titanium(IV) oxide (rutile TiO_2_, Sigma-Aldrich, ≥99.98%), and iridium(IV) oxide (IrO_2_, Sigma-Aldrich, ≥99.9%) were mixed in stoichiometric amounts and ground for 30 min with an agate pestle and mortar for homogeneous mixing of the reagents. A uniaxial press was used to pelletize the mixed powders (∼0.7 g powder material per pellet) then calcined at 900– 1000 °C in air for 12 h, followed by regrinding of the calcined pellets for 30 min before further pelletization and sintering (1000 °C for Ba-based samples, 1400 °C for Sr-based samples) for 12 h in air. A-site deficient samples (Ba_0.9_Ir_0.005_Ti_0.995_O_3_) were synthesized following the same procedure, although calcination and sintering temperatures were 1000 and 1200 °C, respectively.

**Scheme 1:**
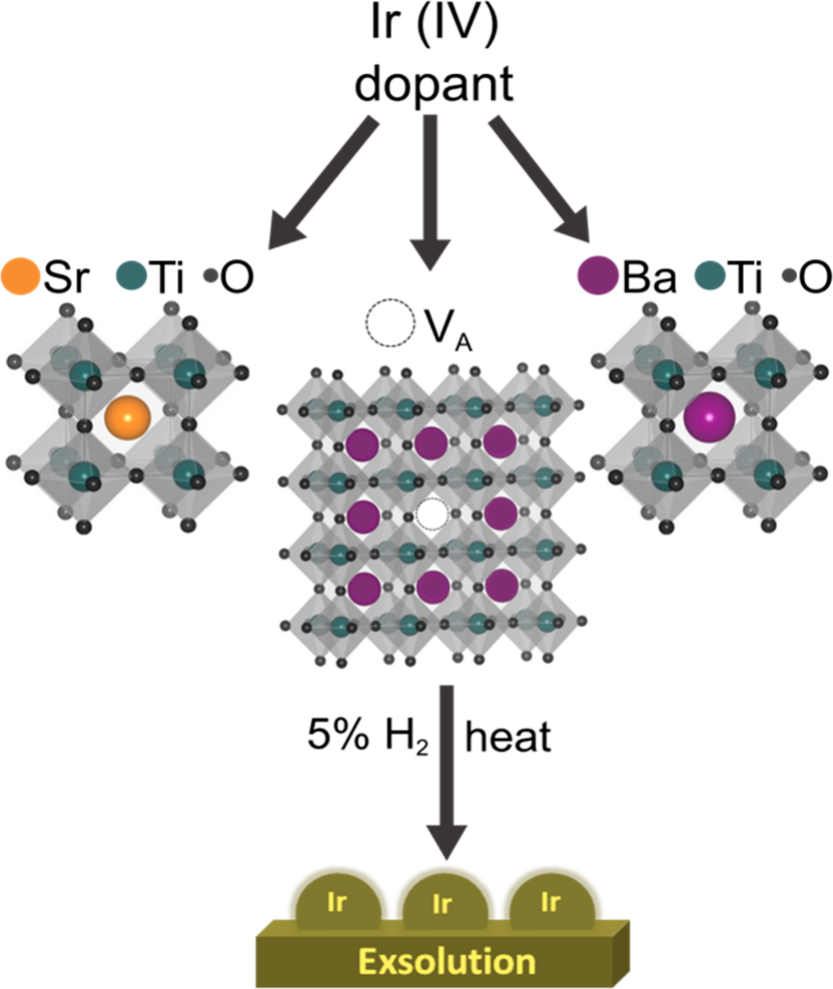
Scheme Illustrating the Structure and Composition of the Ir-Doped Perovskites Described in This Work Undergoing a Controlled Reduction in 5% H_2_/N_2_ Atmosphere Leading to Exsolution of Ir NPs

To obtain exsolution of the as-synthesized samples, reduction of the powder materials was performed in a tube furnace by using a 5% H_2_/Ar flow at either 600 or 900 °C for 10 h and a 5 °C/min heating and cooling rate (Scheme 1).

Benchmark samples were prepared through wet impregnation, using iridium(III) chloride hydrate (IrCl_3_·xH_2_O, Alfa Aesar, 99.9% metals basis), γ-Al_2_O_3_ (puralox scfa-230, Sasol Germany GmbH, 98%), and an undoped BaTiO_3_ sample synthesized through solid-state reaction, as described above. To achieve a nominal 0.4 wt % loading per each impregnated sample, an aqueous solution of each support (γ-Al_2_O_3_ or BaTiO_3_) was prepared, and the appropriate amount of IrCl_3_·xH_2_O was added while stirring the mixture at 85 °C until complete water evaporation and a slurry was obtained. The prepared slurries were then dried at 80 °C for 12 h in air, and finally calcined and reduced through a “direct reduction” method,^31^ by heating the sample with a 10 °C/min heating rate to 400 °C for 2 h in a 5% H_2_/Ar atmosphere, followed by a second heating step at 900 °C for 1 h. The samples were then ramped to room temperature at a 10 °C/min cooling rate.

X-ray diffraction (XRD) analysis was performed using a PAN-analytical X-ray diffractometer (Cu Kα source) to verify the preparation of solid solutions necessary to ensure homogeneous exsolution. X-ray photoelectron spectroscopy (XPS) was carried out on the samples before and after exsolution, and after catalytic testing, to investigate their surface elemental composition and speciation using a Thermo Scientific K-Alpha+ X-ray photoelectron spectrometer operating at a base pressure of 2 × 10^–9^ mbar. This system incorporates a monochromated, microfocused Al Kα X-ray source (*h*ν = 1486.6 eV) and a 180° double-focusing hemispherical analyzer with a 2D detector. The X-ray source was operated at 6 mA emission current and 12 kV anode bias providing an X-ray spot size of up to 400 μm^2^. Core level spectra were recorded at a 20 eV pass energy. A flood gun was used to minimize the sample charging that occurs when an insulated sample is exposed to an X-ray beam. The quantitative XPS analysis was performed using Thermo Avantage software. The binding energy (B.E.) was corrected by aligning the C 1*s* peak of the adventitious carbon (C–C) at 284.8 eV. The intensities of the spectra were normalized to the Ti 2*p*_3/2_ components.

The samples’ nanoscale morphologies and chemical compositions were studied with electron microscopy. Scanning electron microscopy (SEM) was performed using a JEOL JSM-7100F SEM, and transmission electron microscopy (TEM) was performed with a JEOL JEM-2100F TEM operating at a 200 kV voltage and equipped with an energy dispersive X-ray spectroscopy (EDX) detector (EDS 80 mm X-Max detector, Oxford Instruments) for chemical analysis.

A continuous fixed bed quartz reactor loaded with 45 mg of catalyst was used to carry out DRM. The activity test was conducted with a total flow rate of 200 mL min^–1^ (CH_4_/CO_2_/N_2_ = 2.5 %:2.5 %:95 %), giving a weight hourly space velocity (WHSV) of 267000 mL/(g_cat_ h). The reaction temperature was varied between 350 and 750 °C in increments of 50 °C, with each temperature being maintained for 20 min. An online gas analyzer (ABB AO2020 Advanced Optima Process Gas Analyzer, ABB, Mannheim, Germany) was employed to monitor the reactants and products, and the gas components analyzed at the outlet reported as measured (%). The equations used for measuring the samples’ performance are provided below:
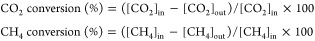
where [CO_2_]_in_ and [CH_4_]_in_ represent molar flow rates of the input CO_2_ and CH_4_, while [CO_2_]_out_ and [CH_4_]_out_ molar flow rates of the output gases. The carbon balance was closed within 5% error for each test in accordance with other published work that was conducted in the same rig.^32^

Carbon deposition measurements were carried out by temperature-programmed oxidation (TPO) using a mass spectrometer (Omni-Star GSD 320) and a secondary electron multiplier detector. In a typical experiment, 45 mg of catalyst were placed in the quartz tube reactor along with quartz wool to secure its position. The samples were oxidized under a 50 mL min^–1^ flow of 3% O_2_ in Ar with a heating ramp from room temperature to 750 °C. The final temperature was chosen based on the main forms of carbonaceous deposits usually observed in the DRM reaction: monatomic/polymeric carbon, detected below 380 °C, “whisker” carbon, analyzed at 440 < *T* < 640 °C, and graphitic carbon, the most stable form, usually observed at ∼650 °C when oxidizing the sample.^33−35^ To verify this, a preliminary TPO test was conducted up to 950 °C, and the results reported in the SI confirm no evidence of carbon deposition for the exsolved samples, even at higher temperatures (Figure S1). Therefore, 750 °C was chosen as the final temperature for these tests, as this was also the highest temperature the materials were exposed to during catalytic testing. The quantification of total carbon was performed by integration of the CO_2_ curves obtained throughout the experiments.

## Results and Discussion

The choice of the perovskite oxide for this study was based on the lower temperatures required to achieve phase purity when using Ba as the A-site element, compared to the archetypal A-site (Sr, in SrTiO_3_), which often also leads to segregation when sintered at the required higher temperatures to achieve a solid solution.^36,37^ The lower calcination and sintering temperatures are also expected to result in smaller crystal grains, with the added benefit of possibly depleting the perovskite support of a higher amount of exsolvable metal (Ir), which is initially homogeneously distributed as a dopant in the bulk structure of the host. Our choice of Ir as the active metal was based on the fact that it has shown remarkable activity in several catalytic reactions even in low concentrations, which implies that our work is of impact to areas of application beyond the one demonstrated in this work.^38^ Nevertheless, iridium catalysts have been proven to be very effective in activating methane at low temperatures hence we believe it is an ideal choice for this study.^39^ Moreover, the choice of a perovskite oxide as support was made as perovskites have emerged as a promising class of support materials in heterogeneous catalysis due to their high thermal stability, structural features, and tunable nature.^40^ In order to attempt to lower the process temperatures further (exsolution temperature, as well as synthesis temperatures), we first studied a slightly A-site deficient material, as this has been proven to be an efficient way of controlling the defect chemistry,^20^ leading to a higher degree of exsolution, while requiring lower temperatures than for stoichiometric materials. With this aim, a Ba_0.9_Ir_0.005_Ti_0.995_O_3-δ_ (namely Ir-B_0.9_TO) was synthesized.

### Correlating the Activity of A-Site Deficient Barium Titanates to Their Exsolution Temperature

**Figure 1:**
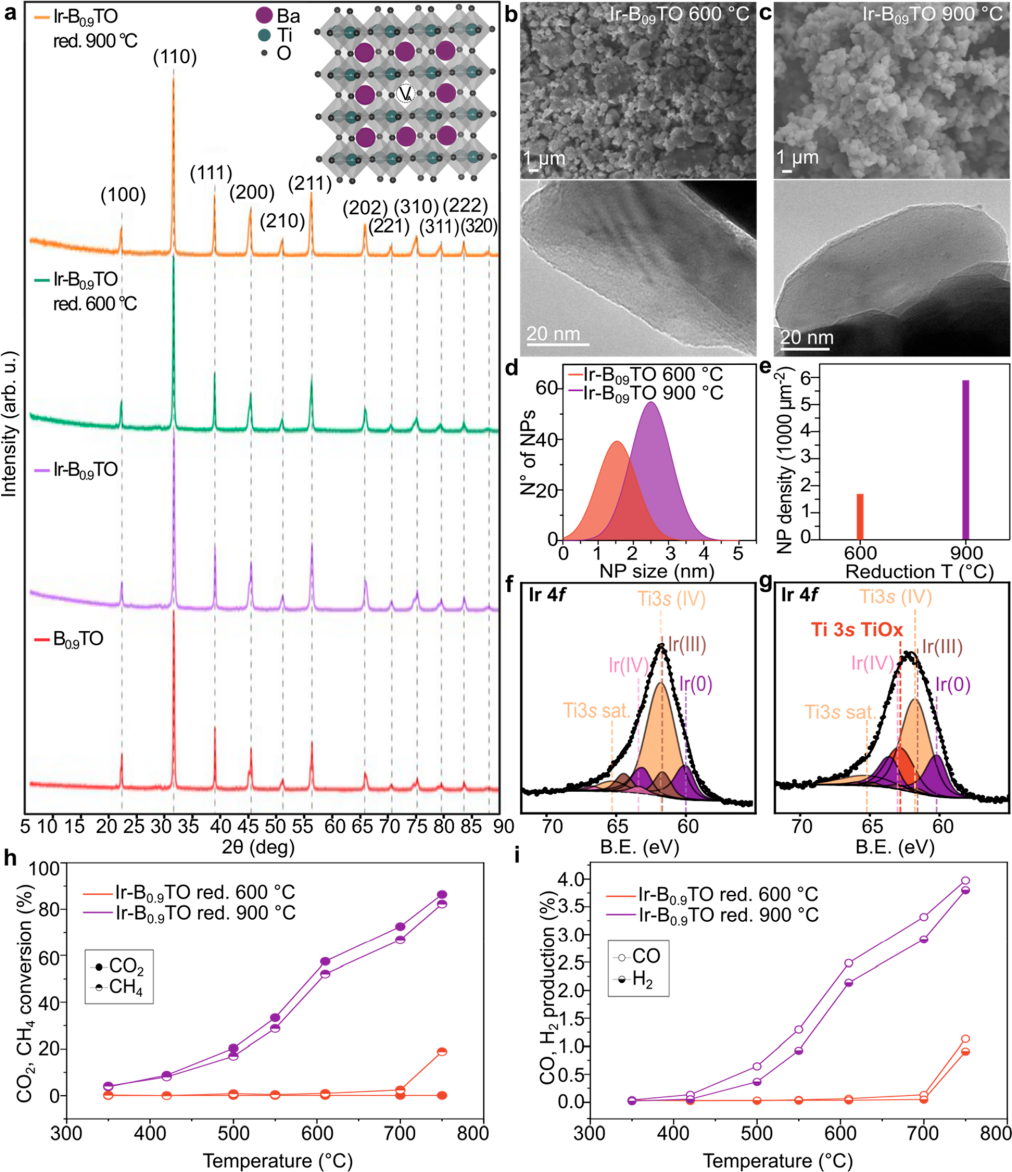
Characterization of the Ir-B_0.9_TO sample series, with (a) XRD patterns showing the characteristic diffraction signals of the BaTiO_3_ crystalline structure for the undoped Ba_0.9_TiO_3_ (red), 0.5% Ir-doped Ba_0.9_TiO_3_ (purple), and 0.5% Ir-doped Ba_0.9_TiO_3_ reduced at 600 °C (green) and at 900 °C (orange), and a schematic representing the synthesized perovskite structure with an A-site vacancy (V_A_) substituting one Ba site. (b, c) SEM (top panel) and TEM (bottom panel) micrographs of the Ir-B_0.9_TO reduced at 600 °C (b) and at 900 °C (c), with corresponding (d) size distribution analysis and (e) population density of the exsolved NPs for the two samples reduced at the different temperatures. (f, g) Ir 4*f* core levels acquired by XPS for the samples reduced at 600 °C (f) and 900 °C (g) showing the Ti^4+^ components in orange, the Ir^3+^ components in brown, the Ir^0^ components in purple, and the additional TiOx component of the Ir-B_0.9_TO sample originating after reduction at 900 °C in dark orange. (h, i) Catalytic activity results comparing the conversion% of CO_2_ and CH_4_ (h) and the CO and H_2_ production% (i) for the two samples.

As the variation of the reduction temperature is a key factor for unlocking the ability to control exsolved particle size and population, as well as the degree of the metal segregation on the oxide surface, the as-synthesized deficient perovskite was reduced at two different temperatures, 600 and 900 °C. Figure 1a shows a comparison of the X-ray diffraction patterns of the A-site deficient barium titanate series. No secondary phase was detected through XRD, suggesting that phase purity was achieved and retained for the whole series. Figure 1b and c shows the morphology (through SEM images) and exsolution (through TEM images) of the samples reduced at the two studied temperatures. Due to the resolution limitations of the technique, SEM was employed for the microstructure study of the materials, revealing a homogeneous size distribution of the perovskite grains. TEM was used to investigate the structure of the exsolved catalysts at the nanoscale and showed the presence of Ir nanoparticles on both reduced samples. Specifically, the Ba_0.9_Ir_0.005_Ti_0.995_O_3-δ_ sample reduced at 600 °C resulted in Ir NPs with an average size of 1.6 ± 0.4 nm (Figure 1d) and a population density of ∼1566 NP μm^–2^, as evaluated by TEM (Figure 1e). The higher reduction temperature resulted in larger NPs on the surface of the host grains, with an average size of 2.5 ± 0.5 nm, as well as a higher NP density (∼5760 NP μm^–2^), as reported in Figure 1d and e. To evaluate the amount of metallic Ir present on the surface after exsolution, XPS analysis was performed, and the Ir 4*f* core level results are reported in Figure 1f and g. The reduction of the sample at 600 °C resulted in a lower amount of Ir^0^ at the surface (Figure 1f) compared to the sample reduced at 900 °C (Figure 1g), 59% to 83%, respectively (Ir^(0)^:Ir^(III),(IV)^ calculated for both samples). This is expected, as the higher reduction temperature would result in a higher degree of reduction of the material as well as the increased mobility of Ir ions toward the surface.^41,42^ As confirmation, a relatively high amount of Ir in its oxide forms is still found in the sample after reduction at 600 °C (Ir^(III),(IV)^:Ti = 0.6%:99.4%), which is lowered (Ir^(III),(IV)^:Ti = 0.2%:99.8%) after reduction in 5% H_2_/Ar at 900 °C for the same time scale (10 h). When the catalytic activity of the two samples for the dry reforming of methane was compared, the sample reduced at 900 °C showed higher activity, as shown in Figure 1h and i. Despite the slightly bigger average size of the exsolved Ir NPs in this sample compared to the ones obtained after reduction at 600 °C, the overall amount of Ir^0^ found at the surface of the reduced Ir-B_0.9_TO at 900 °C is considerably higher and hence likely responsible for its higher activity for this reaction.

### Evaluating the Impact of A-Site Deficiency on Barium Titanates’ Overall Performance

**Figure 2:**
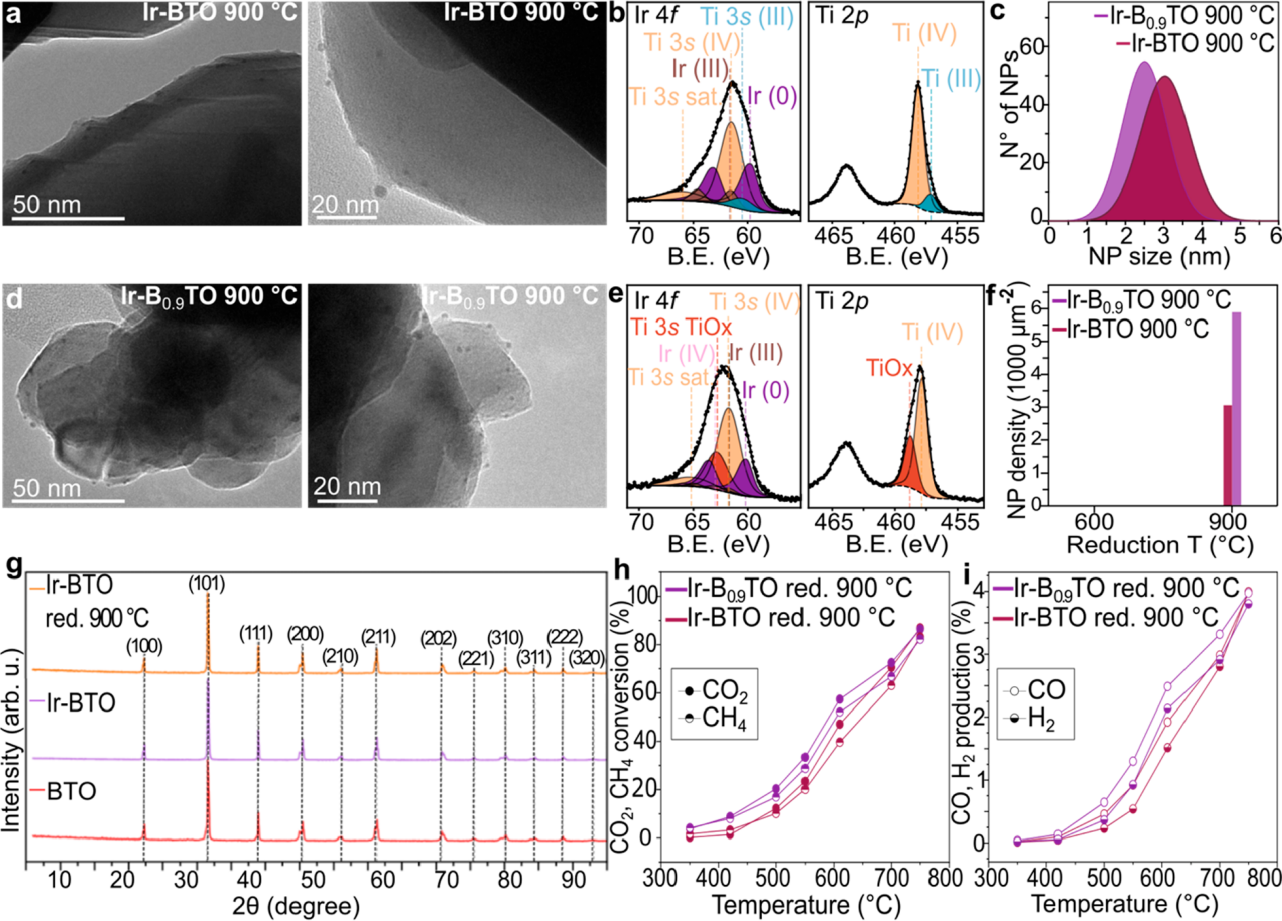
Characterization and catalytic activity of the stoichiometric and A-site deficient Ir-doped barium titanate samples reduced at 900 °C. TEM (a, d) and XPS Ir 4*f* and Ti 2*p* core levels (b, e) of the Ir-BTO and the Ir-B_0.9_TO, respectively, showing the Ti^4+^ components in orange, the Ti^3+^ components in teal, the Ir^3+^ components in brown, the Ir^0^ components in purple, and the additional “TiOx” component of the Ir-B_0.9_TO sample originating after reduction at 900 °C in dark orange. NP (c) size distribution and (f) population density of the two samples compared. (g) XRD patterns of the stoichiometric undoped (red), Ir-doped (purple), and Ir-doped reduced at 900 °C (orange) barium titanate sample showing phase purity for all three samples. (h, i) catalytic activity testing comparing the CO_2_ and CH_4_ conversion% (h) and the CO and H_2_ production% for the two samples compared.

Despite the promising results obtained for the 0.5% Ir-doped B_0.9_TO sample reduced at 900 °C, an extra peak appeared in the Ti 3*s* core level after reduction, which is likely to be due to an ill-defined surface-based TiO_2_ phase, and not present in the sample reduced at 600 °C. This is also visible in the Ti 2*p* core level, where a higher B.E. component at 458.8 eV is only found in this sample, in addition to the one at 457.8 eV attributable to the Ti^4+^ in the Ba_0.9_TiO_3_ lattice. This peak is not observed in the sample reduced at 600 °C (Figure S2 and Tables S1–S3). This therefore suggests a possible surface decomposition/reconstruction of the A-site deficient Ir-doped BTO sample after reduction at 900 °C,^43,44^ as not visible through XRD (or SEM/TEM). This leads us to believe that such a sample might not be stable after prolonged operation under the required reaction conditions. To determine this, we investigated whether, at the same reduction temperature, an A-site stochiometric sample would behave similarly in terms of material’s stability, degree of exsolution, and, consequently, catalytic activity. Figure 2a and d show TEM micrographs of a BaIr_0.005_Ti_0.995_O_3_ stoichiometric sample (Ir-BTO) and of the A-site deficient Ba_0.9_Ir_0.005_Ti_0.995_O_3-δ_ sample (Ir-B_0.9_TO), respectively, both after reduction in 5% H_2_/Ar at 900 °C. The stoichiometric sample also showed exsolved metallic Ir NPs, confirmed by the presence in the Ir 4*f* core level of two intense contributions at 60.0 and 63.1 eV, corresponding to Ir^0^ 4*f*_7/2_ and 4*f*_5/2_ doublets (Figure 2b).

When comparing the Ti 2*p* core levels of the two different samples, the stoichiometric material (Figure 2b) did not show the presence of the TiO_*x*_ surface phase which was indeed visible for the A-site deficient sample (Figure 2e). On the other hand, the Ir-BTO sample showed partial reduction of the Ti in the host structure, from the +4 to +3 oxidation state (Tables S4 and S5 ). This has been previously observed in titanate samples when reduced in similar conditions.^18,45^ When analyzing the average NP size and the surface distribution of the exsolved Ir NPs, the stoichiometric sample displayed slightly larger NPs (3.4 ± 1.1 nm) (Figure 2c), but a considerably lower NP density compared to the Ir-B_0.9_TO sample after reduction at 900 °C (Figure 2f), with 2932 to 5760 NP μm^–2^ measured, respectively. This is not surprising since A-site deficiency was introduced, and is known, to promote exsolution.^19^ Interestingly, however, the measured catalytic activity was found to be comparable for the two exsolved samples, with a ∼90% conversion (CO_2_, CH_4_) obtained with both the 900 °C-reduced Ir-B_0.9_TO and Ir-BTO samples, as seen in Figure 2h. This could be explained by the nonhomogeneous NP distribution when analyzing the surface of the A-site deficient sample, with some grains presenting much lower NP density than others, hence potentially explaining the similar activity observed for both samples. Moreover, considering the application and long-term testing of these samples, Ir-B_0.9_TO decomposition could lead to instability during operation. This has been reported in the majority of the literature studies for similar systems where phase decomposition and exsolution occurred simultaneously;^21,46−48^ hence, Ir-BTO was chosen for further testing and investigation.

### Studying the Influence of the A-Site Chemistry

**Figure 3:**
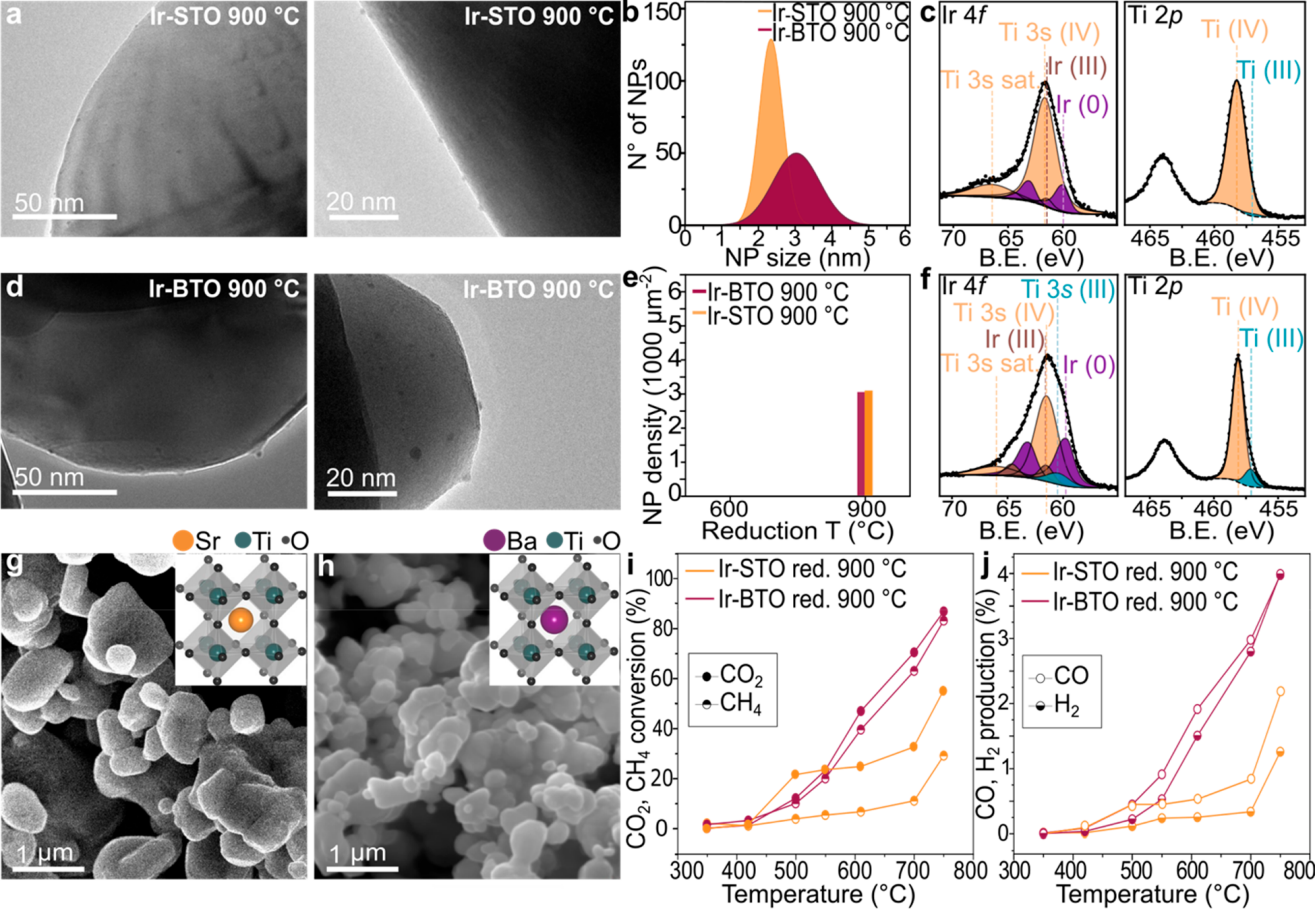
Characterization and catalytic activity comparison of the Ir-STO and Ir-BTO samples reduced at 900 °C. (a) TEM images of the exsolved Ir-STO sample. (b) Exsolved NPs size distribution plot. (c) Ir 4*f* and Ti 2*p* core levels obtained by XPS analysis for the Ir-STO sample showing the Ti^4+^ components in orange, the Ti^3+^ components in teal, the Ir^3+^ components in brown, and the Ir^0^ components in purple. (d) TEM images of the exsolved Ir-BTO sample. (e) NP population graph of the exsolved Ir-STO and the exsolved Ir-BTO samples compared after reduction at 900 °C. (f) Ir 4*f* and Ti 2*p* core levels obtained by XPS analysis for the Ir-BTO sample showing the Ti^4+^ components in orange, the Ti^3+^ components in teal, the Ir^3+^ components in brown, and the Ir^0^ components in purple. (g, h) SEM images showing the grain morphology of the Ir-STO perovskite (g) and of the Ir-BTO perovskite (h), with corresponding insets showing the ABO_3_ crystal structures with the Sr cation (orange) and the Ba cation (purple). (i, j) Catalytic activity testing comparing the CO_2_ and CH_4_ conversion% (i) and the CO and H_2_ production% (j) for the two samples compared in the Results and Discussion.

In order to evaluate whether different A-site alkaline earths would have an effect on the exsolution and/or catalytic activity, the best performing Ba-based titanate (Ir-BTO reduced at 900 °C) was compared with the archetypal SrTiO_3_ perovskite after substitution with the same Ir loading (0.5 atomic % substituting Ti at the B site). The TEM characterization in Figure 3a shows the result of the controlled reduction of a SrIr_0.005_Ti_0.995_O_3_ powder at 900 °C for 10 h (5% H_2_/Ar). When comparing Ir-STO to Ir-BTO reduced in the same conditions (Figure 3d), a lower NP size was observed for Ir-STO, evidenced by the analysis in Figure 3b. A 2.4 ± 0.8 nm NP size distribution was found for the exsolved Sr-based sample compared to a 3.4 ± 1.1 nm average NP size for the Ba-based one, as well as a narrower size distribution. A very similar exsolved NP density was observed at the surface of these two samples (2932 NP μm^–2^ for the Ir-BTO and 2972 NP μm^–2^ for the Ir-STO), as shown in Figure 3e; however, the XPS analysis showed a higher amount of metallic Ir on the surface of the BTO (Figure 3f) with respect to the STO (Figure 3c and Table S6). This can be explained by the different grain sizes obtained when synthesizing the two samples compared here. Specifically, a grain size ranging between 0.5 and 2 μm was generally obtained when synthesizing the Ir-STO sample (Figure 3g), whereas the Ir-BTO sample showed much smaller grains (0.2–0.7 μm average), as visible from Figure 3h. Consequently, the host grain size determines the amount of exsolvable metal diffusing to the surface at the same reducing conditions (10 h at 900 °C, 5% H_2_/Ar), where smaller grains would be depleted of more Ir compared to larger grains, hence explaining the higher Ir metal amount at the surface of the smaller-grain perovskite (Ir-BTO). This is also reflected in the catalytic performances of the two samples. Figure 3i and j clearly highlights the Ir-BTO sample as the best performing, with a CO_2_ and CH_4_ conversion of 87% and 83%, compared to the 56% and 29% obtained for the Ir-STO, due to the higher amount of active phase available on the surface of this Ba-based sample. An interesting feature observed for the Ir-STO sample is the different conversion levels exhibited by CO_2_ and CH_4_. Probably due to the low amount of metal present on the surface of this catalyst (Table S7), the sample was obviously not able to activate and hence convert CH_4_ (similar to the 600 °C-reduced Ir-B_0.9_TO).

### A Comparison of the Optimized Exsolved Ir-BTO with Reference Impregnated Catalysts

**Figure 4:**
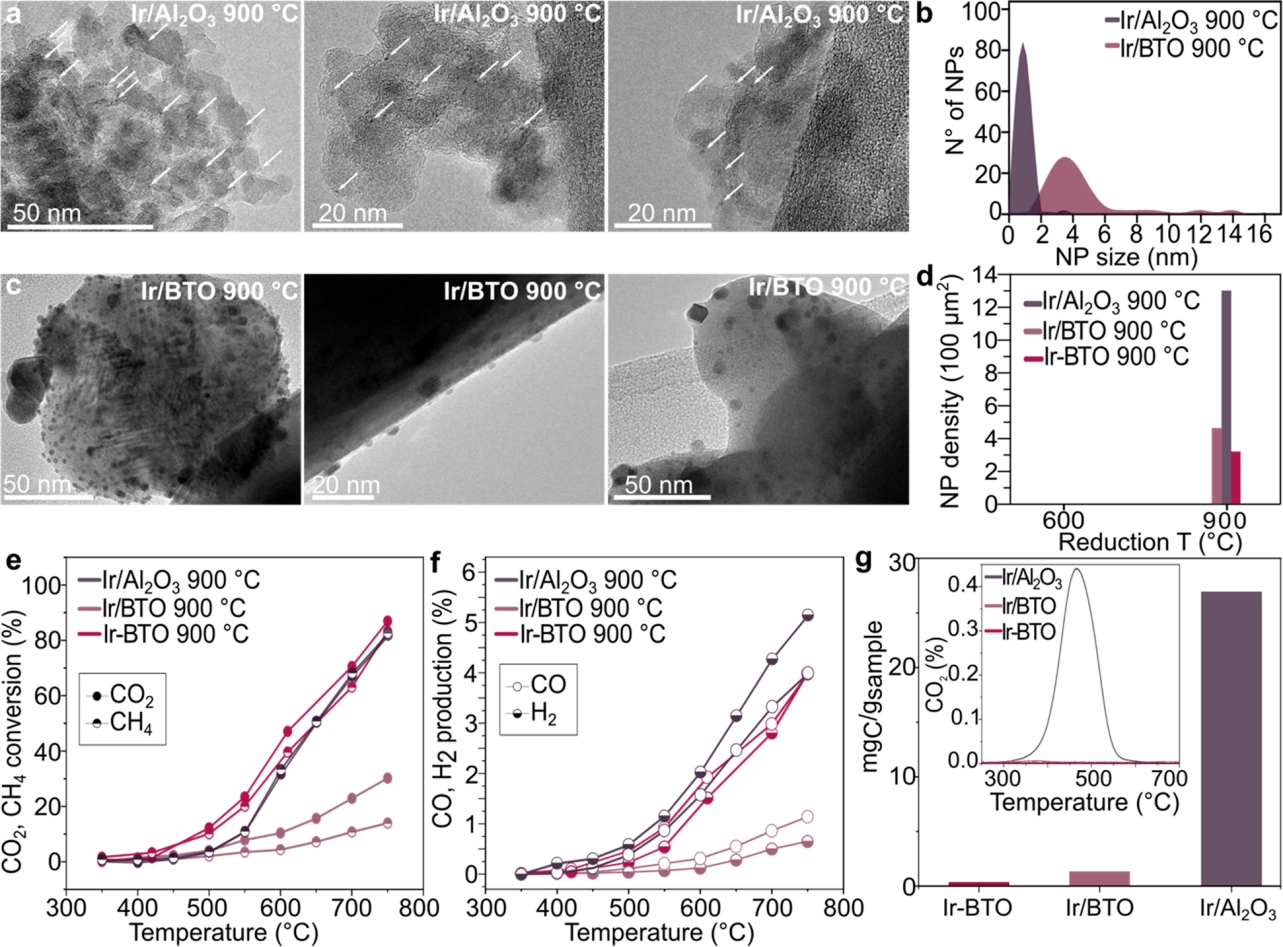
Characterization and catalytic activity comparison of Ir-impregnated Al_2_O_3_ and BTO samples with exsolved Ir-BTO. (a) TEM micrographs of the impregnated Ir/Al_2_O_3_ with arrows indicating some of the impregnated Ir NPs. (b) Impregnated Ir NPs size distribution. (c) TEM micrographs of the impregnated Ir/BTO sample. (d) Population density graph including the comparison with the Ir-BTO sample exsolved at 900 °C. (e, f) Catalytic activity testing comparing the CO_2_ and CH_4_ conversion% (e) and the CO and H_2_ production% (f) for the two samples compared. (g) Carbon deposition quantification after catalytic testing obtained by temperature-programmed oxidation of the two Ir-impregnated samples compared to the one measured for the exsolved Ir-BTO sample, with inset showing the profile of the desorbed CO_2_ over temperature for the three samples.

To evaluate the overall potential of the most active Ir-exsolved sample compared to traditionally used catalytic systems, the sample was benchmarked against two catalysts prepared via wet-impregnation: Ir/BaTiO_3_ and Ir/Al_2_O_3_ powders, on a wt %–wt % loading basis. The 900 °C-reduced impregnated samples showed several differences compared to the exsolved Ir-BTO in terms of micro- and nanostructure, as visible in Figure 4. Looking at the NP size and distribution, the Ir nanoparticles are relatively well-dispersed on Al_2_O_3_, measuring averagely 1.1 ± 0.3 nm (Figure 4a, b), as expected from impregnation on such high-surface-area supports. In comparison, the impregnated Ir/BTO showed a much wider (4.3 ± 2.5 nm) size distribution, as observed from the TEM images in Figure 4c, but a denser surface population, with ∼4360 NP μm^–2^, compared to ∼2932 NP μm^–2^ for the Ir-BTO exsolved at the same temperature (Figure 4d). Both features are not surprising considering the different nature of the methodology employed to obtain surface NPs compared to exsolution. However, controlling the NP size and distribution is nearly impossible in the case of impregnated samples, as indeed visible from Figure 4c. Specifically, when looking at Figure 4b and d, both impregnated samples report higher amounts of NPs on the surface, with the densest population observed for the impregnated Ir/Al_2_O_3_, compared to both the impregnated and exsolved barium titanates. The catalytic activity tests reported in Figure 4e and f, however, showed an interesting outcome, where a much lower conversion is observed for the impregnated BTO-based sample compared with both Ir/Al_2_O_3_ and the exsolved Ir-BTO. Considering that two of the samples have the same support (Ir-BTO and Ir/BTO), we speculate that the strain imposed by the in situ growth of the Ir NPs on the surface of the exsolved sample once again demonstrates the benefits of exsolution to the catalytic activity. When the catalytic activity of the exsolved Ir-BTO is compared with the Ir/Al_2_O_3_ benchmark, an almost identical conversion is observed for both samples. However, a relatively higher H_2_/CO ratio was actually observed for the impregnated Al_2_O_3_-based sample, which indicated that the sample would probably suffer from carbon deposition, ultimately compromising its stability.

To test this hypothesis, we evaluated the level of carbon deposition for these samples after catalytic testing. Looking at the post-test characterization in Figure 4g, it can be noted that C-deposition was negligible for the exsolved Ir-BTO (inset in Figure 4g). However, the benchmark Ir/Al_2_O_3_ showed the presence of carbonaceous deposits after catalytic testing, notoriously the main issue for the dry reforming of methane catalytic reaction, hence suggesting its lack of stability. The impregnated Ir/BTO sample showed instead low C deposition, which was however expected, given the very limited activity shown. Specifically, the stoichiometry of the dry reforming of methane (DRM) dictates that the H_2_/CO ratio should be one. However, when testing the Ir/Al_2_O_3_ catalyst, the ratio was found to exceed unity after reaching a temperature of 550 °C, as verified by the coke formation on this sample. On the other hand, the results for both the Ir-BTO exsolved sample and the Ir/BTO impregnated samples imply resistance to carbon deposition, while, at the same time, it is the prominent parallel reaction (RWGS) that is probably forcing the ratio at values <1 (Figure S3).

### Postcatalytic Test Characterization: The Role of Exsolution in Preventing Sample Degradation

**Figure 5:**
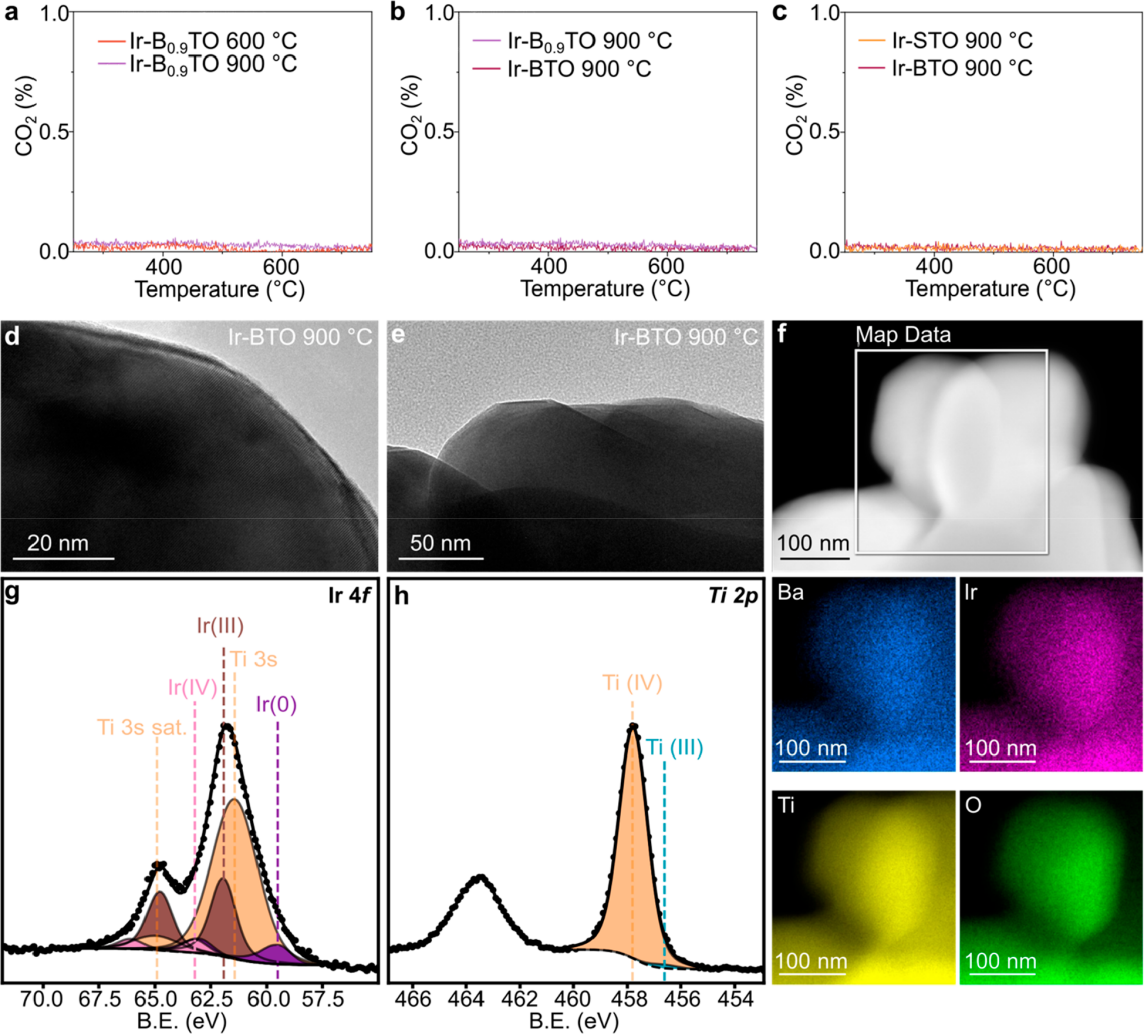
(a–c) Results of the temperature-programmed oxidation carried out on the exsolved samples after their testing for DRM showing no development of CO_2_ for any of the samples. (d, e) TEM images acquired after TPO on the stoichiometric Ir-BTO 900 °C showing absence of exsolved NPs. (f) STEM-EDX elemental maps of Ba, Ir, Ti, and O acquired on grains of the Ir-BTO 900 °C sample after TPO. The signal intensity detected in the Ir color map confirms Ir reincorporation in the perovskite structure. (g) Ir 4*f* and (h) Ti 2*p* XPS core levels showing reoxidation of the Ir-BTO 900 °C sample, with Ir(III), Ir(IV), and Ti(IV) mainly detected after TPO.

Due to the interesting results observed in Figure 4g, we evaluated whether the superior anchorage and strain characteristics obtained through exsolution had an effect in preventing coking, and so we performed TPO measurements, after catalytic testing, on all the other exsolved samples described in this work. As seen in Figure 5a–c, intriguingly, no CO_2_ was measured after temperature-controlled oxidation in 5% O_2_ from RT to 750 °C for any of the exsolved samples, confirming no C deposition had occurred, demonstrating the stability of the catalyst NPs obtained through the exsolution approach. The reason for the high coking resistance of these samples can be explained by the unique morphology of the metal–support interface, where the crystallographic alignment of the two has been shown to alter the carbon deposition mechanism^18,49^ and enhance their stability. Additionally, our results suggest that such features are also responsible for avoiding the proven detrimental effect of the high amount of alkaline earth element on the A-site, which has previously been stated to be a commonly unavoidable issue.^4,17,30^

Moreover, TEM and XPS characterization carried out after TPO showed a remarkable scenario. As visible from Figure 5d–f, when analyzing the stoichiometric Ir-BTO sample previously exsolved at 900 °C, this was found to be NP-free on the surface (Figure 5d, e). This would either mean that the exsolved metallic Ir NPs would undergo reoxidation, with also further volatilization, or that reincorporation of the metal would occur after the oxidation cycle. To study this further, STEM-EDX elemental analysis (Figure 5f) and XPS (Figure 5g, h) were carried out to investigate the possible reincorporation (and change in oxidation state) of Ir in the host. Remarkably, Ir was detected in the (bulk) analyzed area by STEM-EDX, which was mainly found in the +3 and +4 oxidation states by XPS, with only a minor metallic contribution detected after TPO (Table S8). All other exsolved samples were also analyzed after TPO, and reincorporation of the previously exsolved Ir NPs in the host lattice was confirmed for the stoichiometric samples (Figure S4 and Table S9). In contrast, several surface NPs were still found in the analyzed areas of the A-site deficient samples (Figure S5), as expected. Moreover, a lower total Ir content was also measured by XPS, suggesting that a certain degree of volatilization had occurred for these samples after TPO (Figure S6 and Table S9).

**Figure 6:**
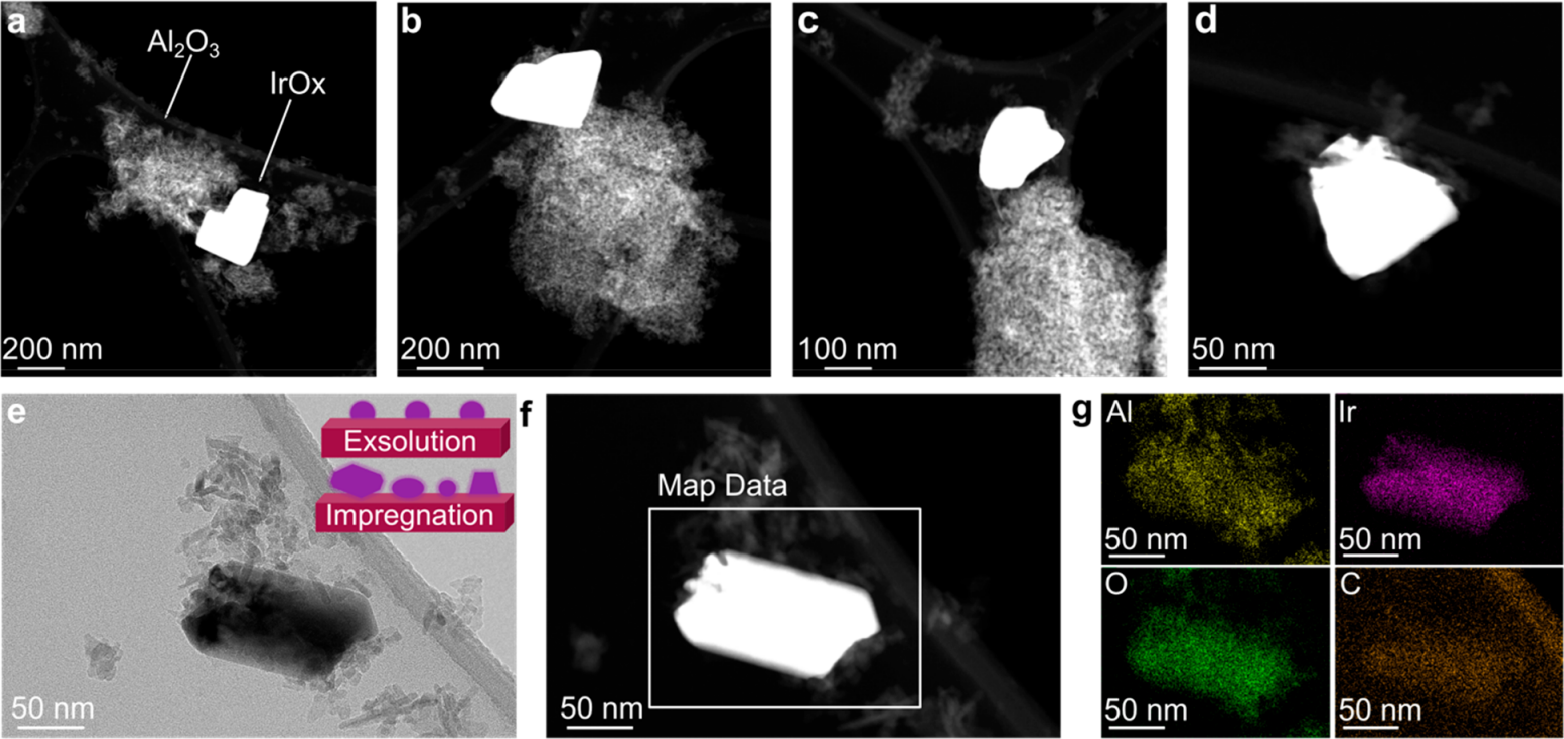
STEM, TEM, and EDX characterizations of the impregnated Ir/Al_2_O_3_ sample after postcatalytic testing TPO. (a-d) HAADF-STEM images showing the bright ∼100–300 nm-sized Ir clusters over the lower-contrast Al_2_O_3_. (e) TEM and (f) STEM images showing the sample region where an EDX map was acquired over a cluster. The schematics in (e) represent the different stability observed for the exsolved sample, where the NPs are “locked” in position due to their socketing, compared to the impregnated benchmark, where extensive coarsening is observed. (g) Elemental maps showing the Ir-O nature of the observed clusters, with the Al map in yellow, Ir map in purple, O map in green, and C map in brown, showing the background C detected over the analyzed region due to the C-film coating of the TEM grid.

For comparison, impregnated Ir/Al_2_O_3_ was also investigated at the nanoscale after TPO, and the results are shown in Figure 6. The impregnated sample showed extensive agglomeration of Ir throughout (bright clusters observed in Figure 6a–d) with Ir clusters ranging from 100 nm (Figure 6e) to 300 nm in size, hence demonstrating the high mobility of Ir when impregnated on such support. STEM-EDX mapping also confirmed the oxidized nature of such Ir clusters, as shown in Figure 6f,g.

## Conclusions

This detailed study of exsolved Ir nanocatalysts for the catalytic conversion of CO_2_ and CH_4_ highlights the key role of exsolution in obtaining high activity while avoiding coking and sintering for reforming reactions. A systematic study of the effect of the exsolution temperature, A-site deficiency, and A-site chemistry has been performed to understand the role of each parameter in the performance of the developed materials for DRM activation. The tested materials were all active for the DRM reaction, with the best-performing system being a stoichiometric Ir-BaTiO_3_ after reduction at 900 °C for 10 h, which ensured both high structural stability and a high overall catalytic performance. When compared to benchmark counterparts obtained via a traditional impregnation method, the exsolved catalyst demonstrated higher stability to deactivation given by its resistance to the formation of carbonaceous deposits, while significant coking and sintering were both observed for the Ir/Al_2_O_3_ system. When looking at previous studies on Ni exsolution for comparison, the metal loading varies and is typically at least 1 order of magnitude higher (>4 wt % active metal). Examples of studies using ∼2–4 wt % active metal resulted in conversions ranging between 45% and 80% CH_4_ and 70%–93% CO_2_; however, deactivation, mainly due to coke formation, was always observed.^28,39,50,51^ Our system, doped with only 0.4 wt % Ir, showed conversions of 82% CH_4_ and 86% CO_2_, without any coke deposition. Therefore, the use of a very dilute amount of Ir proved to be more effective than using Ni alone or combining Ni with another noble metal. The successful active element reincorporation into the structure obtained after oxidation of the exsolved Ir-BTO material shows the promising potential for the reuse of such catalysts, which might be regenerated and re-employed. The combined desired features of high active metal surface density, superior interaction between the support and the nanoparticles, and high resistance to degradation via coking and sintering, and consequently deactivation, show the great benefits of their use in the catalytic conversion of greenhouse gases into H_2_ and other value-added products. Hence, the exsolution method has been proven to be effective in producing strained, stable, and highly active supported nanoparticle catalysts, as confirmed by previous studies and the findings of this paper. It is also of great interest because it allows for the production of tailor-made materials in a single step. However, there are still some limitations associated with this method, mainly related to the efficient exsolution of all the doped amounts in the support matrix. This is due to the low surface area of the materials used, which means that future research in this area should focus on how to exsolve from higher surface area matrices. We believe that addressing this challenge will lead to a step change in the industrial utilization of this highly promising method.

## Data Availability

Supporting research data for this article may be accessed at 10.15126/surreydata.900802.

## Supporting Information Available

Complete XPS peak-fitted data for all the exsolved samples described in this work before and after TPO, additional TPO data and TEM micrographs for the Ir-B_0.9_TO samples after TPO, and H_2_:CO ratio plot and quantifications from XPS analyses (Figures S1–S6, Tables S1–S9) (PDF)

## References

[ref1] Methane emissions remained stubbornly high in 2022 even as soaring energy prices made actions to reduce them cheaper than ever, February 2023. IEA. https://www.iea.org/news/methane-emissions-remained-stubbornly-high-in-2022-even-as-soaring-energy-prices-made-actions-to-reduce-them-cheaper-than-ever.

[ref2] Methane emissions. European Commission. https://energy.ec.europa.eu/topics/oil-gas-and-coal/methane-emissions_en.

[ref3] Lavoie J.-M. Review on Dry Reforming of Methane, a Potentially More Environmentally-Friendly Approach to the Increasing Natural Gas Exploitation. Front. Chem. 2014, 2, 92076 10.3389/fchem.2014.00081. PMC4227528 25426488

[ref4] Arora S.; Prasad R. An Overview on Dry Reforming of Methane: Strategies to Reduce Carbonaceous Deactivation of Catalysts. RSC Adv. 2016, 6 (110), 108668–108688. 10.1039/C6RA20450C.

[ref5] Polyolefins. Plastics Europe. https://plasticseurope.org/plastics-explained/a-large-family/polyolefins-2/.

[ref6] Information for the Package Leaflet Regarding Ethanol Used as an Excipient in Medicinal Products for Human Use. European Medicines Agency. https://www.ema.europa.eu/en/ethanol.

[ref7] Sustainable Aviation Fuel (SAF). International Civil Aviation Organization. https://www.icao.int/environmental-protection/pages/SAF.aspx.

[ref8] Bychkov V. Yu.; Tyulenin Yu. P.; Firsova A. A.; Shafranovsky E. A.; Gorenberg A. Ya.; Korchak V. N. Carbonization of Nickel Catalysts and Its Effect on Methane Dry Reforming. Applied Catalysis A: General 2013, 453, 71–79. 10.1016/j.apcata.2012.12.006.

[ref9] Akri M.; Zhao S.; Li X.; Zang K.; Lee A. F.; Isaacs M. A.; Xi W.; Gangarajula Y.; Luo J.; Ren Y.; Cui Y.-T.; Li L.; Su Y.; Pan X.; Wen W.; Pan Y.; Wilson K.; Li L.; Qiao B.; Ishii H.; Liao Y.-F.; Wang A.; Wang X.; Zhang T. Atomically Dispersed Nickel as Coke-Resistant Active Sites for Methane Dry Reforming. Nat. Commun. 2019, 10 (1), 5181 10.1038/s41467-019-12843-w. 31729358 PMC6858327

[ref10] Joo S.; Seong A.; Kwon O.; Kim K.; Lee J. H.; Gorte R. J.; Vohs J. M.; Han J. W.; Kim G. Highly Active Dry Methane Reforming Catalysts with Boosted in Situ Grown Ni-Fe Nanoparticles on Perovskite via Atomic Layer Deposition. Science Advances 2020, 6 (35), eabb1573 10.1126/sciadv.abb1573. 32923635 PMC7449676

[ref11] Wang J.; Fu Y.; Kong W.; Li S.; Yuan C.; Bai J.; Chen X.; Zhang J.; Sun Y. Investigation of Atom-Level Reaction Kinetics of Carbon-Resistant Bimetallic NiCo-Reforming Catalysts: Combining Microkinetic Modeling and Density Functional Theory. ACS Catal. 2022, 12 (8), 4382–4393. 10.1021/acscatal.2c00027.

[ref12] Bian Z.; Das S.; Wai M. H.; Hongmanorom P.; Kawi S. A Review on Bimetallic Nickel-Based Catalysts for CO_2_ Reforming of Methane. ChemPhysChem 2017, 18 (22), 3117–3134. 10.1002/cphc.201700529. 28710875

[ref13] Maina S. C. P.; Ballarini A. D.; Vilella J. I.; de Miguel S. R. Study of the Performance and Stability in the Dry Reforming of Methane of Doped Alumina Supported Iridium Catalysts. Catal. Today 2020, 344, 129–142. 10.1016/j.cattod.2018.11.023.

[ref14] Postole G.; Nguyen T.-S.; Aouine M.; Gélin P.; Cardenas L.; Piccolo L. Efficient Hydrogen Production from Methane over Iridium-Doped Ceria Catalysts Synthesized by Solution Combustion. Applied Catalysis B: Environmental 2015, 166–167, 580–591. 10.1016/j.apcatb.2014.11.024.

[ref15] Kim Y.; Kim H. S.; Kang D.; Kim M.; Lee J. W. Enhanced Redox Performance of LaFeO_3_ Perovskite through In-Situ Exsolution of Iridium Nanoparticles for Chemical Looping Steam Methane Reforming. Chemical Engineering Journal 2023, 468, 143662 10.1016/j.cej.2023.143662.

[ref16] Yentekakis I. V.; Panagiotopoulou P.; Artemakis G. A Review of Recent Efforts to Promote Dry Reforming of Methane (DRM) to Syngas Production via Bimetallic Catalyst Formulations. Applied Catalysis B: Environmental 2021, 296, 120210 10.1016/j.apcatb.2021.120210.

[ref17] Aramouni N. A. K.; Touma J. G.; Tarboush B. A.; Zeaiter J.; Ahmad M. N. Catalyst Design for Dry Reforming of Methane: Analysis Review. Renewable and Sustainable Energy Reviews 2018, 82, 2570–2585. 10.1016/j.rser.2017.09.076.

[ref18] Neagu D.; Oh T.-S.; Miller D. N.; Ménard H.; Bukhari S. M.; Gamble S. R.; Gorte R. J.; Vohs J. M.; Irvine J. T. S. Nano-Socketed Nickel Particles with Enhanced Coking Resistance Grown in Situ by Redox Exsolution. Nat. Commun. 2015, 6 (1), 8120 10.1038/ncomms9120. 26360910 PMC4579408

[ref19] Kousi K.; Tang C.; Metcalfe I. S.; Neagu D. Emergence and Future of Exsolved Materials. Small 2021, 17 (21), 2006479 10.1002/smll.202006479. 33787009

[ref20] Neagu D.; Tsekouras G.; Miller D. N.; Ménard H.; Irvine J. T. S. In Situ Growth of Nanoparticles through Control of Non-Stoichiometry. Nat. Chem. 2013, 5 (11), 916–923. 10.1038/nchem.1773. 24153368

[ref21] Neagu D.; Irvine J. T. S.; Wang J.; Yildiz B.; Opitz A. K.; Fleig J.; Wang Y.; Liu J.; Shen L.; Ciucci F.; Rosen B. A.; Xiao Y.; Xie K.; Yang G.; Shao Z.; Zhang Y.; Reinke J.; Schmauss T. A.; Barnett S. A.; Maring R.; Kyriakou V.; Mushtaq U.; Tsampas M. N.; Kim Y.; O’Hayre R.; Carrillo A. J.; Ruh T.; Lindenthal L.; Schrenk F.; Rameshan C.; Papaioannou E. I.; Kousi K.; Metcalfe I. S.; Xu X.; Liu G. Roadmap on Exsolution for Energy Applications. J. Phys. Energy 2023, 5 (3), 031501 10.1088/2515-7655/acd146.

[ref22] Kyriakou V.; Sharma R. K.; Neagu D.; Peeters F.; De Luca O.; Rudolf P.; Pandiyan A.; Yu W.; Cha S. W.; Welzel S.; van de Sanden M. C. M.; Tsampas M. N. Plasma Driven Exsolution for Nanoscale Functionalization of Perovskite Oxides. Small Methods 2021, 5 (12), 2100868 10.1002/smtd.202100868. 34928018

[ref23] Khalid H.; Haq A. u.; Alessi B.; Wu J.; Savaniu C. D.; Kousi K.; Metcalfe I. S.; Parker S. C.; Irvine J. T. S.; Maguire P.; Papaioannou E. I.; Mariotti D. Rapid Plasma Exsolution from an A-Site Deficient Perovskite Oxide at Room Temperature. Adv. Energy Mater. 2022, 12 (45), 2201131 10.1002/aenm.202201131.

[ref24] Myung J.; Neagu D.; Miller D. N.; Irvine J. T. S. Switching on Electrocatalytic Activity in Solid Oxide Cells. Nature 2016, 537 (7621), 528–531. 10.1038/nature19090. 27548878

[ref25] Kousi K.; Neagu D.; Bekris L.; Papaioannou E. I.; Metcalfe I. S. Endogenous Nanoparticles Strain Perovskite Host Lattice Providing Oxygen Capacity and Driving Oxygen Exchange and CH4 Conversion to Syngas. Angew. Chem., Int. Ed. 2020, 59 (6), 2510–2519. 10.1002/anie.201915140. 31804017

[ref26] Deka D. J.; Kim J.; Gunduz S.; Aouine M.; Millet J.-M. M.; Co A. C.; Ozkan U. S. Investigation of Hetero-Phases Grown via in-Situ Exsolution on a Ni-Doped (La,Sr)FeO_3_ Cathode and the Resultant Activity Enhancement in CO2 Reduction. Applied Catalysis B: Environmental 2021, 286, 119917 10.1016/j.apcatb.2021.119917.

[ref27] Xiao Y.; Xie K. Active Exsolved Metal-Oxide Interfaces in Porous Single-Crystalline Ceria Monoliths for Efficient and Durable CH_4_/CO_2_ Reforming. Angew. Chem. 2022, 134 (1), e202113079 10.1002/ange.202113079. 34676642

[ref28] Ali S. A.; Safi M.; Merkouri L.-P.; Soodi S.; Iakovidis A.; Duyar M. S.; Neagu D.; Reina T. R.; Kousi K. Engineering Exsolved Catalysts for CO_2_ Conversion. Frontiers in Energy Research 2023, 11, na 10.3389/fenrg.2023.1150000.

[ref29] Falcon H. Double Perovskite Oxides A2FeMoO6?? (A = Ca, Sr and Ba) as Catalysts for Methane Combustion. Applied Catalysis B: Environmental 2004, 53 (1), 37–45. 10.1016/j.apcatb.2004.05.004.

[ref30] Li X.; Hu Q.; Yang Y.; Wang Y.; He F. Studies on Stability and Coking Resistance of Ni/BaTiO3-Al2O3 Catalysts for Lower Temperature Dry Reforming of Methane (LTDRM). Applied Catalysis A: General 2012, 413–414, 163–169. 10.1016/j.apcata.2011.11.004.

[ref31] Nikolaraki E.; Goula G.; Panagiotopoulou P.; Taylor M. J.; Kousi K.; Kyriakou G.; Kondarides D. I.; Lambert R. M.; Yentekakis I. V. Support Induced Effects on the Ir Nanoparticles Activity, Selectivity and Stability Performance under CO_2_ Reforming of Methane. Nanomaterials 2021, 11 (11), 2880 10.3390/nano11112880. 34835645 PMC8624188

[ref32] le Saché E.; Pastor-Pérez L.; Haycock B. J.; Villora-Picó J. J.; Sepúlveda-Escribano A.; Reina T. R. Switchable Catalysts for Chemical CO2 Recycling: A Step Forward in the Methanation and Reverse Water-Gas Shift Reactions. ACS Sustainable Chem. Eng. 2020, 8 (11), 4614–4622. 10.1021/acssuschemeng.0c00551.

[ref33] Großmann K.; Dellermann T.; Dillig M.; Karl J. Coking Behavior of Nickel and a Rhodium Based Catalyst Used in Steam Reforming for Power-to-Gas Applications. Int. J. Hydrogen Energy 2017, 42 (16), 11150–11158. 10.1016/j.ijhydene.2017.02.073.

[ref34] Parizotto N. V.; Rocha K. O.; Damyanova S.; Passos F. B.; Zanchet D.; Marques C. M. P.; Bueno J. M. C. Alumina-Supported Ni Catalysts Modified with Silver for the Steam Reforming of Methane: Effect of Ag on the Control of Coke Formation. Applied Catalysis A: General 2007, 330, 12–22. 10.1016/j.apcata.2007.06.022.

[ref35] Sasson Bitters J.; He T.; Nestler E.; Senanayake S. D.; Chen J. G.; Zhang C. Utilizing Bimetallic Catalysts to Mitigate Coke Formation in Dry Reforming of Methane. Journal of Energy Chemistry 2022, 68, 124–142. 10.1016/j.jechem.2021.11.041.

[ref36] Van Den Bosch C. A. M.; Cavallaro A.; Moreno R.; Cibin G.; Kerherve G.; Caicedo J. M.; Lippert T. K.; Doebeli M.; Santiso J.; Skinner S. J.; Aguadero A. Revealing Strain Effects on the Chemical Composition of Perovskite Oxide Thin Films Surface, Bulk, and Interfaces. Adv. Mater. Interfaces 2020, 7 (2), 1901440 10.1002/admi.201901440.

[ref37] Koo B.; Kim K.; Kim J. K.; Kwon H.; Han J. W.; Jung W. Sr Segregation in Perovskite Oxides: Why It Happens and How It Exists. Joule 2018, 2 (8), 1476–1499. 10.1016/j.joule.2018.07.016.

[ref38] Wang C.; Yang F.; Feng L. Recent Advances in Iridium-Based Catalysts with Different Dimensions for the Acidic Oxygen Evolution Reaction. Nanoscale Horizons 2023, 8 (9), 1174–1193. 10.1039/D3NH00156C. 37434582

[ref39] Martin R.; Kim M.; Asthagiri A.; Weaver J. F. Alkane Activation and Oxidation on Late-Transition-Metal Oxides: Challenges and Opportunities. ACS Catal. 2021, 11 (8), 4682–4703. 10.1021/acscatal.1c00612.

[ref40] Zhu J.; Li H.; Zhong L.; Xiao P.; Xu X.; Yang X.; Zhao Z.; Li J. Perovskite Oxides: Preparation, Characterizations, and Applications in Heterogeneous Catalysis. ACS Catal. 2014, 4 (9), 2917–2940. 10.1021/cs500606g.

[ref41] Calì E.; Kerherve G.; Naufal F.; Kousi K.; Neagu D.; Papaioannou E. I.; Thomas M. P.; Guiton B. S.; Metcalfe I. S.; Irvine J. T. S.; Payne D. J. Exsolution of Catalytically Active Iridium Nanoparticles from Strontium Titanate. ACS Appl. Mater. Interfaces 2020, 12 (33), 37444–37453. 10.1021/acsami.0c08928. 32698571

[ref42] Calì E.; Thomas M. P.; Vasudevan R.; Wu J.; Gavalda-Diaz O.; Marquardt K.; Saiz E.; Neagu D.; Unocic R. R.; Parker S. C.; Guiton B. S.; Payne D. J. Real-Time Insight into the Multistage Mechanism of Nanoparticle Exsolution from a Perovskite Host Surface. Nat. Commun. 2023, 14 (1), 1754 10.1038/s41467-023-37212-6. 36990982 PMC10060596

[ref43] Barzilay M.; Qiu T.; Rappe A. M.; Ivry Y. Epitaxial TiOx Surface in Ferroelectric BaTiO_3_: Native Structure and Dynamic Patterning at the Atomic Scale. Adv. Funct. Mater. 2020, 30 (18), 1902549 10.1002/adfm.201902549.

[ref44] O’Reilly T.; Holsgrove K. M.; Zhang X.; Scott J. J. R.; Gaponenko I.; Kumar P.; Agar J.; Paruch P.; Arredondo M. The Effect of Chemical Environment and Temperature on the Domain Structure of Free-Standing BaTiO_3_ via In Situ STEM. Advanced Science 2023, 10, 2303028 10.1002/advs.202303028. 37607120 PMC10582436

[ref45] Psiuk B.; Szade J.; Pilch M.; Szot K. XPS Studies of Perovskites Surface Instability Caused by Ar+ Ion and Electron Bombardment and Metal Deposition. Vacuum 2009, 83, S69–S72. 10.1016/j.vacuum.2009.01.032.

[ref46] Dimitrakopoulos G.; Ghoniem A. F.; Yildiz B. In Situ Catalyst Exsolution on Perovskite Oxides for the Production of CO and Synthesis Gas in Ceramic Membrane Reactors. Sustainable Energy Fuels 2019, 3 (9), 2347–2355. 10.1039/C9SE00249A.

[ref47] Chen X.; Ni W.; Wang J.; Zhong Q.; Han M.; Zhu T. Exploration of Co-Fe Alloy Precipitation and Electrochemical Behavior Hysteresis Using Lanthanum and Cobalt Co-Substituted SrFeO_3-δ_ SOFC Anode. Electrochim. Acta 2018, 277, 226–234. 10.1016/j.electacta.2018.05.019.

[ref48] Cao X.; Ke L.; Zhao K.; Yan X.; Wu X.; Yan N. Surface Decomposition of Doped PrBaMn_2_O_5+δ_ Induced by In Situ Nanoparticle Exsolution: Quantitative Characterization and Catalytic Effect in Methane Dry Reforming Reaction. Chem. Mater. 2022, 34 (23), 10484–10494. 10.1021/acs.chemmater.2c02488.

[ref49] Umar A.; Neagu D.; Irvine J. T. S. Alkaline Modified A-Site Deficient Perovskite Catalyst Surface with Exsolved Nanoparticles and Functionality in Biomass Valorisation. Biofuel Research Journal 2021, 8 (1), 1342–1350. 10.18331/BRJ2021.8.1.5.

[ref50] Yao X.; Cheng Q.; Attada Y.; Ould-Chikh S.; Ramírez A.; Bai X.; Mohamed H. O.; Li G.; Shterk G.; Zheng L.; Gascon J.; Han Y.; Bakr O. M.; Castaño P. Atypical Stability of Exsolved Ni-Fe Alloy Nanoparticles on Double Layered Perovskite for CO_2_ Dry Reforming of Methane. Applied Catalysis B: Environmental 2023, 328, 122479 10.1016/j.apcatb.2023.122479.

[ref51] Chava R.; Seriyala A. K.; Varma D B. A.; Yeluvu K.; Roy B.; Appari S. Investigation of Ba Doping in A-Site Deficient Perovskite Ni-Exsolved Catalysts for Biogas Dry Reforming. Int. J. Hydrogen Energy 2023, 48 (71), 27652–27670. 10.1016/j.ijhydene.2023.03.464.

