# Supplementary material for: Enhanced Stability of Iridium Nanocatalysts via Exsolution for the CO2 Reforming of Methane
Source: ACS Appl Nano Mater. 2023 Dec 1;7(16):18398–409. doi: 10.1021/acsanm.3c04126 (PMC11348315; doi:10.1021/acsanm.3c04126)
Supplement: Supplementary file 1 — an3c04126_si_001.pdf [file an3c04126_si_001.pdf]

Supporting Information

Enhanced Stability of Iridium Nanocatalysts via Exsolution for the CO_2 Reforming of Methane

Eleonora Calì[1,2], Shailza Saini[3], Gwilherm Kerherve[2], William S. Skinner[2], Ian S. Metcalfe[4], David J. Payne[2,5], Kalliopi Kousi[3]**

[1] Department of Applied Science and Technology, Politecnico di Torino, Corso Duca degli Abruzzi, 24, Turin 10129, Italy.

[2] Department of Materials, Imperial College London, Exhibition Road, London SW7 2AZ, United Kingdom

[3] School of Chemistry and Chemical Engineering, University of Surrey, Guildford, GU2 7XH, United Kingdom

[4] School of Engineering, Newcastle University, Merz Court, Newcastle upon Tyne NE1 7RU, United Kingdom

[5] Research Complex at Harwell, Harwell Science and Innovation Campus, Didcot, Oxfordshire OX11 0FA, United Kingdom

Email: eleonora.cali@polito.it; k.kousi@surrey.ac.uk

Supplementary Data

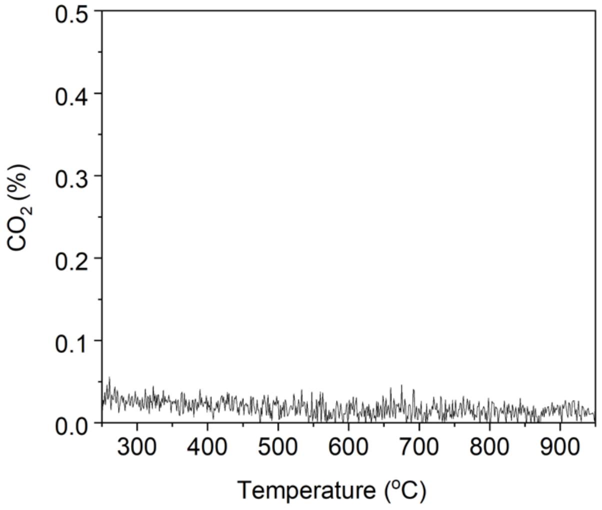

Figure S1. Example of preliminary TPO tests for this work conducted up to 950 °C confirming no C deposition occurring even at higher temperatures on the exsolved samples.

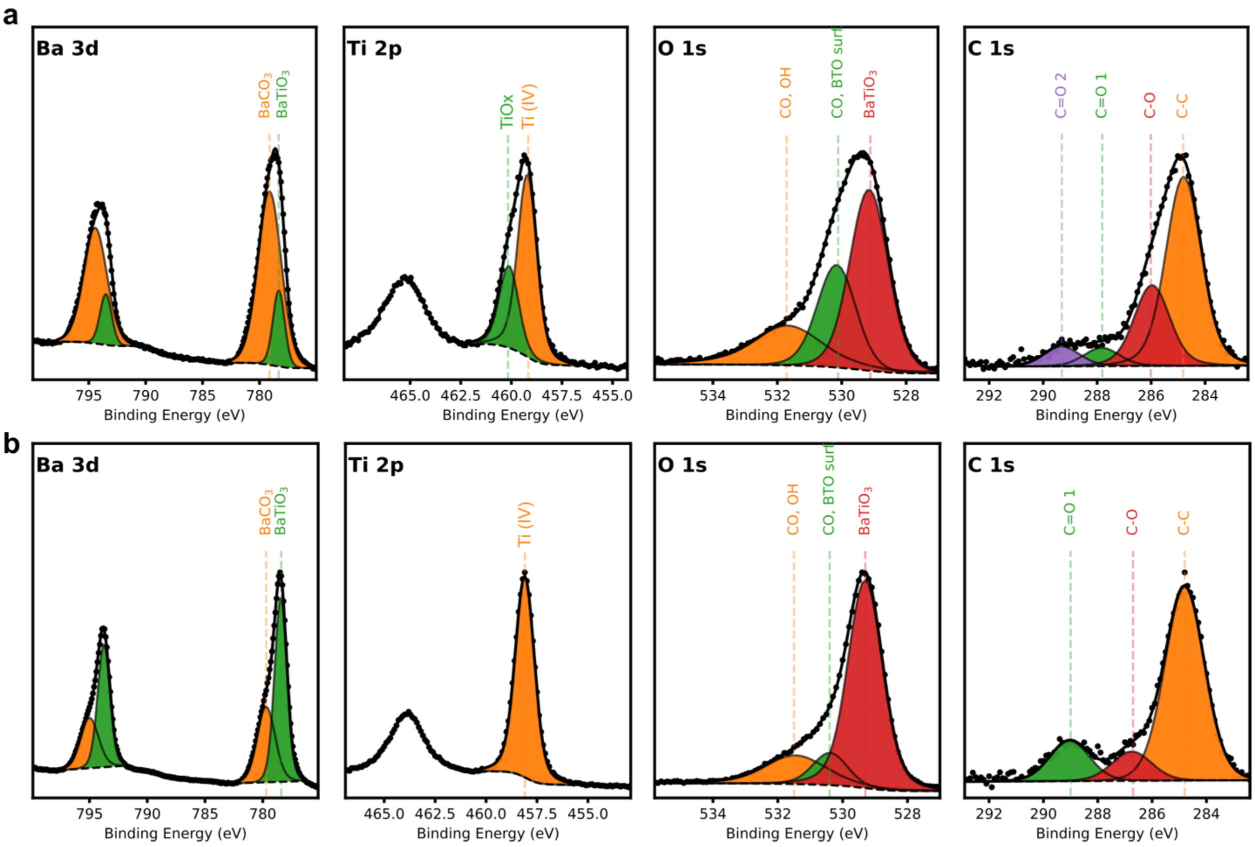

Figure S2. XPS data of the A-site deficient Ir-$B_{0.9}$TO reduced at 900 °C **(a)** and 600 °C **(b)** with all the peak-fitted core levels not included in Figure 1 of the main document: Ba 3*d*, Ti 2*p*, O 1*s*,

C 1*s*. The TiOx component attributed to the sample degradation is only found in the Ti 2*p* peak fitted core level of the sample reduced at 900 °C, and not in the one reduced at 600 °C.

Table S1. Quantification obtained from the XPS analysis of the Ir-$B_{0.9}TO$ sample reduced at 600 °C (Figure S1).

Name	Peak BE	FWHM eV	Atomic %
Ba $3d_{5/2}$ $BaCO_3$	779.7	1.86	6.13
Ba $3d_{5/2}$ $BaTiO_3$	778.44	1.27	10.19
Ir $4f_{7/2}$ Ir(0)	60.01	1.68	0.16
Ir $4f_{7/2}$ Ir(III)	61.66	1.4	0.09
Ir $4f_{7/2}$ Ir(IV)	63.39	1.4	0.02
O1s O-lattice	529.3	1.26	45.27
O1s O-surface	530.39	1.26	7.04
O 1*s* $BaCO_3$, OH	531.46	2.24	11.88
Ti $2p_{3/2}$ Ti(IV)	458.09	1.12	19.22
Ti$2p_{3/2}$ Ti(III)	456.12	1.11	0.0

Table S2. Quantification obtained from the XPS analysis of the Ir-$B_{0.9}TO$ sample reduced at 900 °C (Figure S1).

**Name**	**Peak BE**	**FWHM eV**	**Atomic %**
Ba $3d_{5/2}$ $BaCO_3$	779.11	2.46	13.18
Ba $3d_{5/2}$ $BaTiO_3$	778.3	1.22	2.87
Ir $4f_{7/2}$ Ir(0)	60.15	1.78	0.2
Ir $4f_{7/2}$ Ir(III)	61.67	1.4	0.02

Ir $4f_{7/2}$ Ir(IV)	63.0	1.4	0.02
O1s O-lattice	529.15	1.41	32.19
O1s O-surface, O-Ti	530.16	1.41	18.24
O $1s$ $BaCO_3$, OH	531.68	2.54	14.4
Ti $2p_{3/2}$ Ti(IV)	457.85	1.13	13.01
Ti $2p_{3/2}$ TiO_2	458.76	1.13	5.86

Table S3. Comparison of elemental ratios obtained from the XPS analyses of the Ir-$B_{0.9}$TO sample reduced at 600 °C and 900 °C (Figure S1).

	**Ir-$B_{0.9}$TO 600 °C**	**Ir-$B_{0.9}$TO 900 °C**
Ba $3d_{5/2}$ $BaTiO_3$: Ir $4f_{7/2}$ Total : O $1s$ (BTO+surf) : Ti $2p_{3/2}$ (IV+III)	12:4 : 0.33 : 63.8 : 23.4	4.3 : 0.4 : 75.8 : 19.5
Ir $4f_{7/2}$ Total : Ti $2p_{3/2}$ (IV+III)	1.4 : 98.6	1.8 : 98.2
Ba $3d_{5/2}$ $BaTiO_3$: Ti $2p_{3/2}$ (IV+III)	34.6 : 65.4	18.1 : 81.9
Ir $4f_{7/2}$ Ir(IV) : Ir(III) : Ir(0)	7.4 : 33.3 : 59.3	8.3 : 8.3 : 83.4
Ti $2p_{3/2}$ Ti(IV) : Ti(III)	100 : 0	100 : 0
Ti $2p_{3/2}$ Ti(IV+III) : TiO_2	100 : 0	68.9 : 31.1

Table S4. Quantification obtained from the XPS analysis of the Ir-BTO sample reduced at 900 °C.

Name	Peak BE	FWHM eV	Atomic %
Ba 3$d_{5/2}$ $BaCO_3$	780.03	1.4	3.06
Ba 3$d_{5/2}$ $BaTiO_3$ 1	778.63	1.4	13.51
Ba 3$d_{5/2}$ $BaTiO_3$ 2	777.48	1.4	1.37
Ir 4$f_{7/2}$ Ir(0)	59.72	2	0.29
Ir 4$f_{7/2}$ Ir(III)	61.52	1.5	0.07
Ir 4$f_{7/2}$ Ir(IV)	--	--	0
O1s O-lattice	529.28	1.43	43.15
O1s O-surface	530.79	1.43	8.8
O1s CO, OH/O-Si	532.13	1.71	13.84
Ti 2$p_{3/2}$ Ti(III)	457.13	1.14	2.01
Ti 2$p_{3/2}$ Ti(IV)	458.13	1.14	13.92

Table S5. Comparison of elemental ratios obtained from the XPS analyses of the Ir-BTO and Ir-$B_{0.9}$TO samples reduced at 900 °C (Figure 2).

	**IrBTO 900 °C**	**Ir$B_{0.9}$TO 900 °C**
Ba 3$d_{5/2}$ $BaTiO_3$: Ir 4$f_{7/2}$ Total : O 1s (BTO+surf) : Ti 2$p_{3/2}$ (IV+III)	20.0 : 0.5 : 58.0 : 21.4	4.3 : 0.4 : 75.8 : 19.5
Ir 4$f_{7/2}$ Total : Ti 2$p_{3/2}$ (IV+III)	2.2 : 98.8	1.8 : 98.2
Ba 3$d_{5/2}$ $BaTiO_3$: Ti 2$p_{3/2}$ (IV+III)	48.3 : 51.7	18.1 : 81.9
Ir 4$f_{7/2}$ Ir(IV) : Ir(III) : Ir(0)	0 : 19.4 : 80.6	8.3 : 8.3 : 83.4
Ti 2$p_{3/2}$ Ti(IV) : Ti(III)	87.4 : 12.6	100 : 0
Ti 2$p_{3/2}$ Ti(IV+III) : TiO_2	100 : 0	68.9 : 31.1

Table S6. Quantification obtained from the XPS analysis of the Ir-STO sample reduced at 900 °C.

Name	Peak BE	FWHM eV	Atomic %
Sr 3$d_{5/2}$ Sr-surface	133.6	1.34	3.05
Sr 3$d_{5/2}$ $SrTiO_3$	132.7	1.44	14.77
Ir 4$f_{7/2}$ Ir(0)	59.95	1.61	0.12
Ir 4$f_{7/2}$ Ir(III)	61.42	1.21	0.03
Ir 4$f_{7/2}$ Ir(IV)	--	--	0
O1s O-lattice	529.11	1.64	26.5
O1s Sr-O	529.81	1.64	19.95
O1s CO, OH	531.6	2.48	19.41
Ti 2$p_{3/2}$ Ti(III)	457.03	1.68	0.33
Ti2p3 Ti(IV)	458.23	1.68	15.84

Table S7. Comparison of elemental ratios obtained from the XPS analyses of the Ir-BTO and Ir-STO samples reduced at 900 °C (Figure 3).

	**IrBTO 900 °C**	**IrSTO 900 °C**
(Ba or Sr) 3$d_{5/2}$ (Ba or Sr)TiO_3 : Ir 4$f_{7/2}$ Total : O 1*s* (BaTO or STO+surf) : Ti 2$p_{3/2}$ (IV+III)	20.0 : 0.5 : 58.0 : 21.4	19.9 : 0.2 :59.9 : 20.9
Ir 4$f_{7/2}$ Total : Ti 2$p_{3/2}$ (IV+III)	2.2 : 98.8	0.9 : 99.1
(Ba or Sr) 3$d_{5/2}$ (Ba or Sr)TiO_3 : Ti 2$p_{3/2}$ (IV+III)	48.3 : 51.7	47.7 : 52.3
Ir 4$f_{7/2}$ Ir(IV) : Ir(III) : Ir(0)	0 : 19.4 : 80.6	0 : 20 : 80

Ti $2p_{3/2}$ Ti(IV) : Ti(III)	87.4 : 12.6	98.0 : 2.0
Ti $2p_{3/2}$ Ti(IV+III) : TiO_2	100 : 0	100 : 0

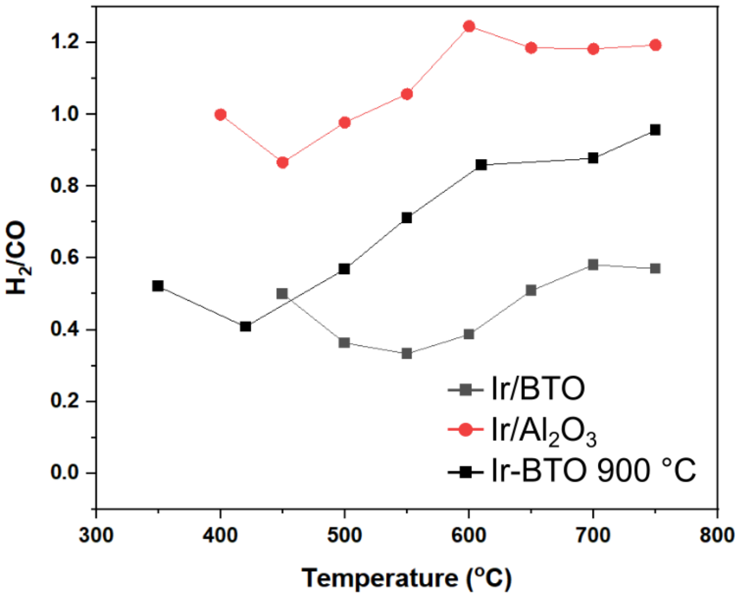

Figure S3. Plot showing the H_2:CO measured for the three samples compared in Figure 4 of the main manuscript document: the exsolved Ir-BTO reduced at 900 °C, the impregnated Ir/BTO, and the impregnated Ir/Al_2O_3.

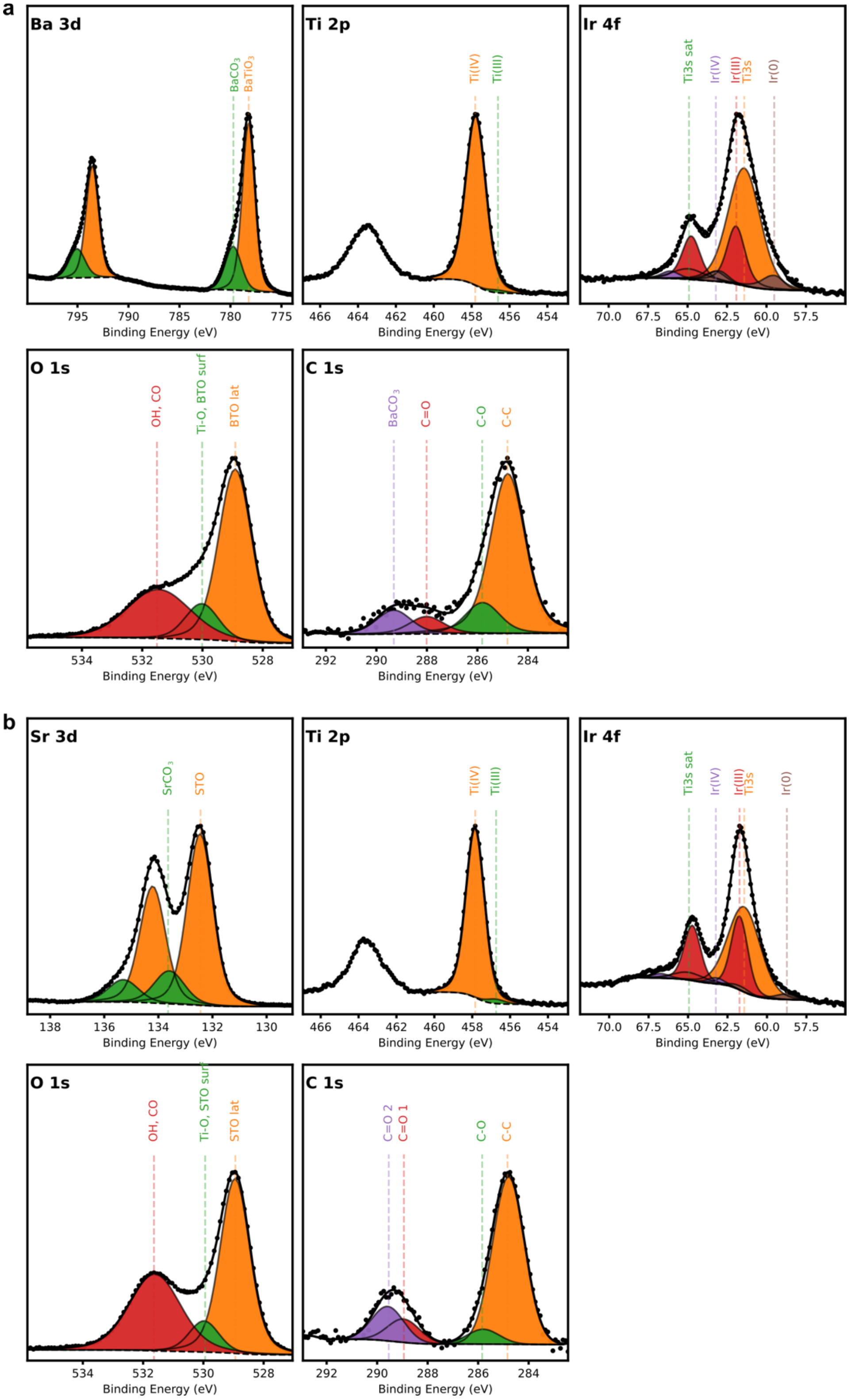
a
Ba 3d
BaCO₃
BaTiO₃
795 790 785 780 775
Binding Energy (eV)
Ti 2p
Ti(IV)
Ti(III)
466 464 462 460 458 456 454
Binding Energy (eV)
Ir 4f
Ti3s sat
Ir(IV)
Ir(III)
Ti3s
Ir(0)
70.0 67.5 65.0 62.5 60.0 57.5
Binding Energy (eV)
O 1s
OH, CO
Ti-O, BTO surf
BTO lat
534 532 530 528
Binding Energy (eV)
C 1s
BaCO₃
C=O
C-O
C-C
292 290 288 286 284
Binding Energy (eV)
b
Sr 3d
SrCO₃
STO
138 136 134 132 130
Binding Energy (eV)
Ti 2p
Ti(IV)
Ti(III)
466 464 462 460 458 456 454
Binding Energy (eV)
Ir 4f
Ti3s sat
Ir(IV)
Ir(III)
Ti3s
Ir(0)
70.0 67.5 65.0 62.5 60.0 57.5
Binding Energy (eV)
O 1s
OH, CO
Ti-O, STO surf
STO lat
534 532 530 528
Binding Energy (eV)
C 1s
C=O 2
C=O 1
C-O
C-C
292 290 288 286 284
Binding Energy (eV)

Figure S4. XPS data of the stoichiometric Ir-BTO **(a)** and Ir-STO **(b)** previously exsolved at 900 °C after TPO. All the peak-fitted core levels are presented: Ba 3*d*, Sr 3*d*, Ti 2*p*, Ir 4*f*, O 1*s*, C 1*s*. The Ir 4*f* for both samples shows the reoxidation of the previously metallic Ir to Ir(III)/Ir(IV), confirming that reincorporation of the previously exsolved metal occurs in these stoichiometric samples after TPO, without sample degradation.

Table S8. Quantification obtained from the XPS analysis of the Ir-BTO sample reduced at 900 °C after TPO.

**Name**	**Peak BE**	**FWHM eV**	**Atomic %**
Ba $3d_{5/2}$ $BaCO_3$	779.73	1.87	4.59
Ba $3d_{5/2}$ $BaTiO_3$	778.22	1.49	14.08
Ir $4f_{7/2}$ Ir(0)	59.5	1.63	0.06
Ir $4f_{7/2}$ Ir(III)	61.93	1.36	0.2
Ir $4f_{7/2}$ Ir(IV)	63.2	1.36	0.03
O1s O-lattice	528.91	1.3	37.26
O1s O-surface	530.02	1.3	7.76
O1s CO, OH	531.46	2.49	20.3
Ti $2p_{3/2}$ Ti(III)	456.66	1.26	0.25
Ti $2p_{3/2}$ Ti(IV)	457.78	1.26	15.47

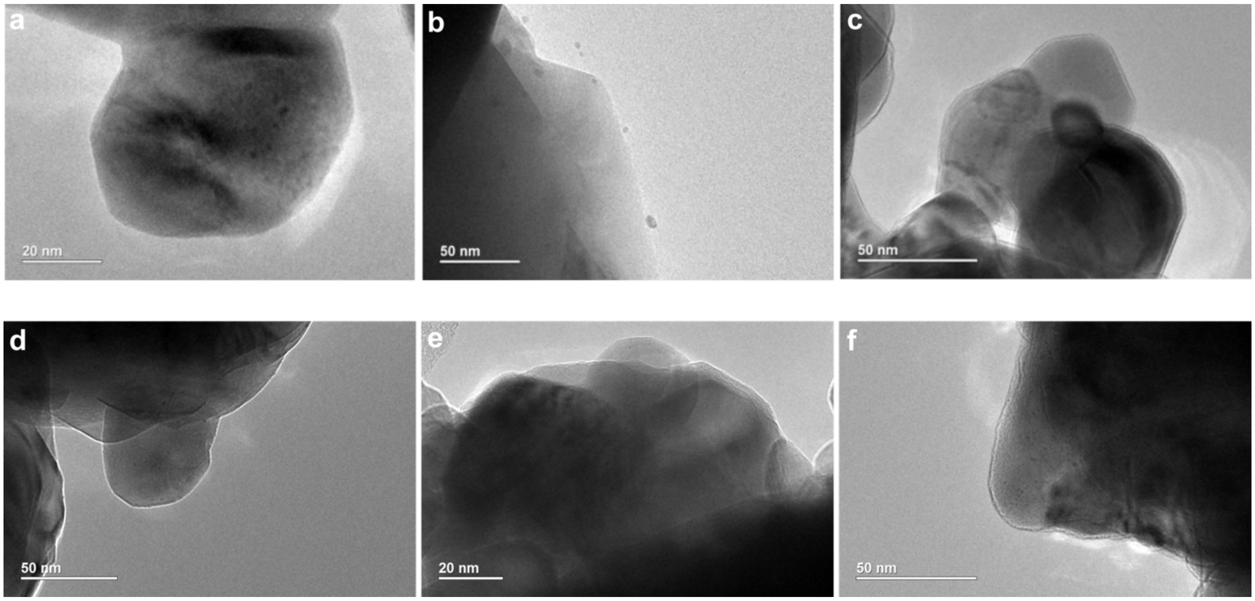

Figure S5. TEM images showing the presence of several surface NPs on the A-site deficient Ir-$B_{0.9}$TO previously reduced at 900 °C (a-c) and at 600 °C (d-f) after TPO, suggesting exsolution is not fully reversible in these compositions.

Table S9. Comparison of elemental ratios obtained from the XPS analyses of the exsolved samples described in this work after TPO.

	**IrBTO 900 °C TPO**	**Ir$B_{0.9}$TO 600 °C TPO**	**Ir$B_{0.9}$TO 900 °C TPO**	**IrSTO 900 °C TPO**
(Ba or Sr) $3d_{5/2}$ (Ba or Sr)TiO_3 : Ir $4f_{7/2}$ Total : O 1*s* (BaTO or STO+surf) : Ti $2p_{3/2}$ (IV+III)	18.7 : 0.4 : 59.9 : 20.9	14.4 : 0.12 : 62.4 : 23.1	14.6 : 0.12 : 60.9 : 24.4	20.3 : 0.4 : 59.9 : 19.4
(Ba or Sr) $3d_{5/2}$ (Ba or Sr)TiO_3 : Ti $2p_{3/2}$ (IV+III)	47.2 : 52.8	38.4 : 61.6	37.5 : 62.5	51.2 : 48.8
Ir $4f_{7/2}$ Ir(IV) :	10.3 : 69.0 :	10.0 : 60.0 : 30.0	20 : 40 : 40	7.7 : 84.6 : 7.7

Ir(III) : Ir(0)	20.7			
Ti $2p_{3/2}$ Ti(IV) : Ti(III)	98.4 : 1.6	99.0 : 1.0	97.8 : 2.2	97.4 : 2.6
Ti $2p_{3/2}$ Ti(IV+III) : TiO_2	100 : 0	100 : 0	100 : 0	100 : 0

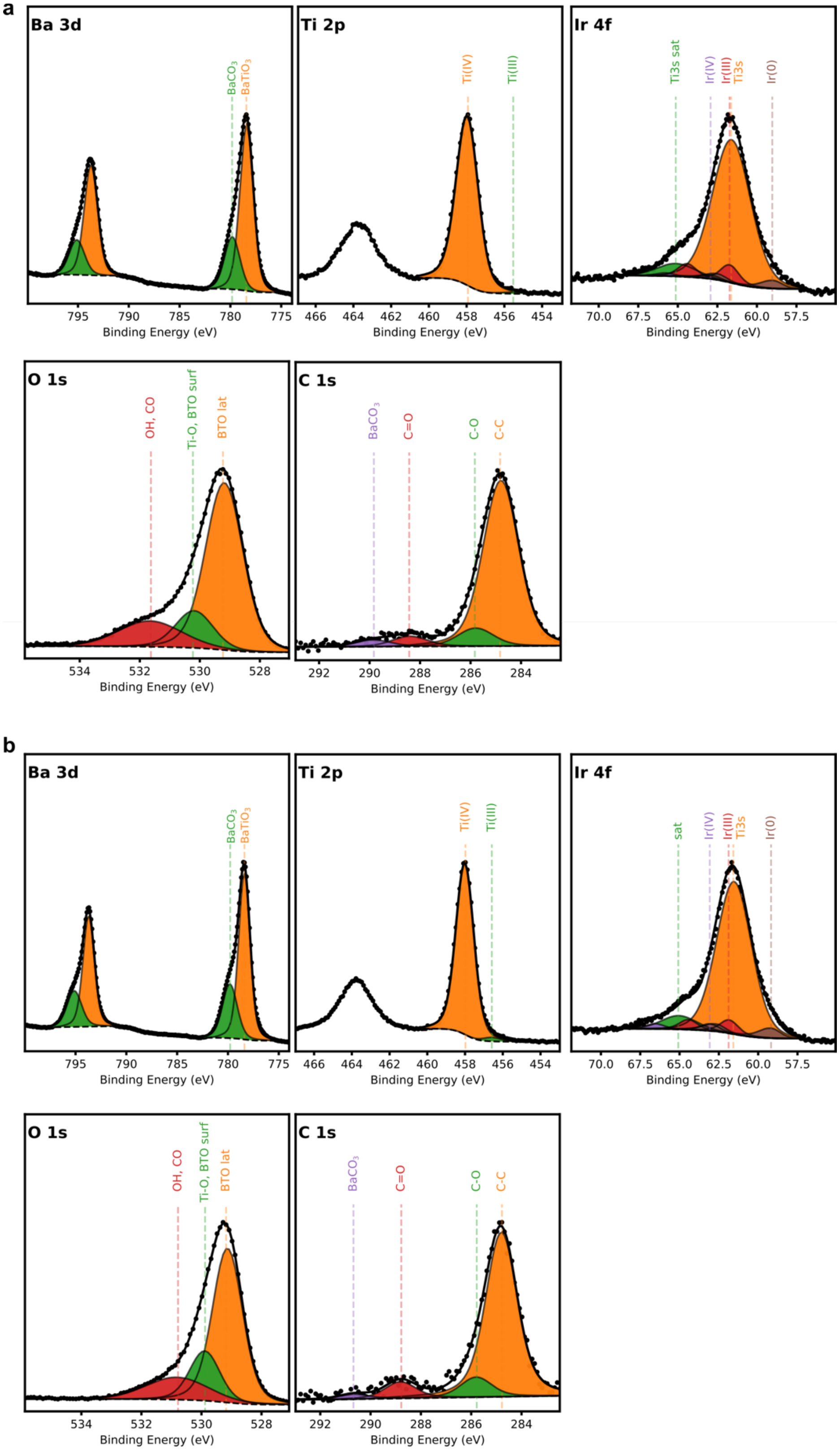
a
Ba 3d
BaCO3
BaTiO3
795
790
785
780
775
Binding Energy (eV)
Ti 2p
Ti(IV)
Ti(III)
466
464
462
460
458
456
454
Binding Energy (eV)
Ir 4f
Ti3s sat
Ir(IV)
Ir(III)
Ti3s
Ir(0)
70.0
67.5
65.0
62.5
60.0
57.5
Binding Energy (eV)
O 1s
OH, CO
Ti-O, BTO surf
BTO lat
534
532
530
528
Binding Energy (eV)
C 1s
BaCO3
C=O
C-O
C-C
292
290
288
286
284
Binding Energy (eV)
b
Ba 3d
BaCO3
BaTiO3
795
790
785
780
775
Binding Energy (eV)
Ti 2p
Ti(IV)
Ti(III)
466
464
462
460
458
456
454
Binding Energy (eV)
Ir 4f
sat
Ir(IV)
Ir(III)
Ti3s
Ir(0)
70.0
67.5
65.0
62.5
60.0
57.5
Binding Energy (eV)
O 1s
OH, CO
Ti-O, BTO surf
BTO lat
534
532
530
528
Binding Energy (eV)
C 1s
BaCO3
C=O
C-O
C-C
292
290
288
286
284
Binding Energy (eV)

Figure S6. XPS data of the A-site deficient Ir-$B_{0.9}$TO previously reduced at 600 °C **(a)** and 900 °C **(b)** after TPO with all the peak-fitted core levels (Ba 3*d*, Ti 2*p*, Ir 4*f*, O 1*s*, C 1*s*). The TiOx component attributed to the sample degradation previously found in the Ti 2*p* peak-fitted core level of the sample reduced at 900 °C is not visible after TPO. However, for both samples, the amount of Ir is found diminished, and the Ir(0) component is still evident, specifically in the Ir 4*f* core level of the sample previously exsolved at 900 °C.
